# New Horizons of Biomarkers in Metastatic Thyroid Cancer

**DOI:** 10.7150/jca.101395

**Published:** 2025-01-01

**Authors:** Alicia Sanchez Cendra, Leonel Pekarek, Beatriz Iglesias Pedrejón, Loreto Bernier, Eduardo David Roberts Cervantes, Cristina Sanchez Cendra, Javier Cassinello, Laura López-Gonzalez, Félix Mañez, Concepción Blanco Carrero, Clara Tasende Fernández, Tomás Ratia Giménez, Diego Martín Córdova García, Alberto Gutiérrez-Calvo, Melchor Álvarez-Mon, Raúl Díaz-Pedrero, Miguel A Ortega

**Affiliations:** 1 Oncology Service, Guadalajara University Hospital, 19002 Guadalajara, Spain.; 2 Department of Surgery, Medical and Social Sciences, Faculty of Medicine and Health Sciences, University of Alcalá, 28801 Alcala de Henares, Spain.; 3 Ramón y Cajal Institute of Sanitary Research (IRYCIS), 28034 Madrid, Spain; 4 Department of General and Digestive Surgery. General and Digestive Surgery. Hospital Universitario Príncipe de Asturias. 28805 Alcalá de Henares, Madrid, Spain; 5 Deparment of Medicine and Medical Specialities (CIBEREHD), Faculty of Medicine and Health Sciences, University of Alcalá. 28801 Alcalá de Henares, Spain; 6 Endocrinology and Nutrition Service, Hospital Univer-sitario Príncipe de Asturias (CIBEREHD), 28806, Alcalá de Henares, Spain; 7 Inmune System Diseases-Rheumatology and Internal medicine Service. Hospital Univer-sitario Príncipe de Asturias (CIBEREHD), 28806, Alcalá de Henares, Spain

**Keywords:** Thyroid Cancer, Biomarkers, Diagnosis, Prognosis, Treatment, Personalized Medicine

## Abstract

This review focuses on the latest advancements in using biomarkers to diagnose, predict outcomes, and guide the treatment of different types of thyroid cancer, such as anaplastic, papillary, medullary, and follicular thyroid carcinoma. We highlight the key role of both traditional and new biomarkers in improving the treatment of these cancers. For anaplastic thyroid cancer, biomarkers are crucial for detecting distant metastases and making treatment decisions. In papillary thyroid carcinoma, biomarkers are used to predict patient survival, taking into account factors like age, the presence of metastasis, and the extent of the tumor. Medullary thyroid carcinoma has seen advancements due to genetic research, particularly in identifying RET mutations, which help in selecting targeted treatments. In follicular thyroid carcinoma, understanding the tumor's molecular profile is important for assessing its aggressiveness and potential to spread. The review also discusses the therapeutic use and variability of various biomarkers in advanced thyroid cancers. Among the most relevant innovations are advancements in detection techniques such as the detection of circulating tumor DNA (ctDNA) and microRNA is emerging as a promising tool for monitoring the disease and predicting treatment response. The integration of these biomarkers into clinical practice, including the most recent detection techniques, is a significant step toward personalized medicine, leading to more accurate diagnoses and better outcomes for thyroid cancer patients

## 1. Introduction

The evolving landscape of thyroid cancer treatment is witnessing significant advancements due to the integration of established and emerging biomarkers. This progress is notably impacting the diagnosis, treatment, and prognosis of various types of thyroid cancer, including anaplastic thyroid cancer (ATC), papillary thyroid carcinoma (PTC), medullary thyroid carcinoma (MTC), and follicular thyroid carcinoma (FTC).

Regarding the epidemiology of thyroid cancer, it can be divided according to histological presentation. Firstly, anaplastic thyroid cancer, although rare, accounting for less than 5% of thyroid cancers, is notable for its aggressive behavior and predominantly affects older adults, generally over 60 years of age, with a slight female predominance. The median survival is around 6 months after diagnosis. The rapid progression and poor prognosis of this subtype demand urgent clinical attention and research 1,2. In contrast, papillary thyroid carcinoma is the most common form, constituting approximately 80-85% of thyroid cancer cases. It is more frequently diagnosed in women, typically between the ages of 30 to 50, and is characterized by a generally favorable prognosis. However, aggressive variants of PTC present significant challenges in treatment and outcome prediction 3,4. Adding complexity to thyroid cancers, medullary thyroid carcinoma (MTC) represents about 2-3% of these cancers. Originating from the parafollicular C-cells of the thyroid, MTC can occur both sporadically and as part of hereditary syndromes. Its management is heavily influenced by genetic factors, especially mutations in the RET proto-oncogene, underscoring the importance of genetic insights in its treatment 5,6. Finally, follicular thyroid carcinoma arises from the thyroid's follicular cells. While it represents a smaller portion of thyroid cancers, FTC is significant for its potential for lymphovascular invasion and progression to metastatic disease. The clinical behavior of FTC varies, ranging from minimally to widely invasive forms, which significantly impacts its prognosis and therapeutic approaches 7.

It is important to highlight that several biomarker detection techniques are offering promising results in current studies and are expected to be implemented in clinical practice in the future. These techniques include the detection of circulating tumor DNA (ctDNA), microRNA, spatial transcriptomics (SPT), and single-cell RNA sequencing (scRNA-seq).

The integration of biomarkers into thyroid cancer management heralds a significant shift towards more precise and personalized treatment strategies. The diversity in epidemiology and prognosis across thyroid cancer subtypes, ranging from the aggressive and challenging ATC to the more common and treatable PTC, underscores the complexity of this disease spectrum. The crucial role of genetic factors in MTC and the invasive nature of FTC further emphasize the need for individualized approaches in treatment planning. As we continue to expand our understanding and application of both established and emerging biomarkers, the prospects for improved diagnostic accuracy, treatment efficacy, and patient outcomes in thyroid cancer care become increasingly attainable. This progress not only reflects the dynamic nature of thyroid cancer research but also highlights the need for ongoing innovation and exploration in this field.

## 2. Papillary Thyroid Cancer

Papillary Thyroid Cancer (PTC) stands as the most prevalent type of thyroid malignancy, constituting approximately 80-85% of all thyroid cancer cases. PTC is distinguished by its indolent nature and excellent prognosis, with most patients experiencing a 5-year survival rate exceeding 95%. This favorable outcome is largely due to the typically slow growth rate of PTC and its responsiveness to established treatments, including surgical resection, radioactive iodine ablation, and thyroid hormone therapy. We should highlight that its higher incidence is among women and is most frequently diagnosed in individuals in their 30s and 40s, though it can occur at any age 8. Despite its generally favorable prognosis, the clinical course of PTC can vary significantly based on tumor size, histological characteristics, the presence of lymph node or distant metastases, and patient age at diagnosis. Certain histological variants of PTC, such as tall cell, columnar, and hobnail variants, are associated with a more aggressive behavior and may necessitate more comprehensive therapeutic approaches 9. The identification and integration of biomarkers specific to PTC have become crucial for refining diagnostic accuracy, tailoring treatment plans, and enhancing prognostic assessments. These biomarkers, including genetic mutations such as BRAF V600E and rearrangements like RET/PTC, not only offer insights into the pathogenesis of PTC but also open avenues for targeted therapy options.

This section of the article explores the different role of diverse biomarkers in PTC that would allow deeper understanding of molecular mechanisms in the line to advance towards more effective and individualized patient care.

### 2.1. Histological Biomarkers

The diagnosis of papillary thyroid carcinoma is based on architectural features along with nuclear clearing, overlap, grooves, and pseudoinclusions. When this papillary architecture is absent, distinguishing follicular variant papillary thyroid carcinoma from cellular adenomatous nodules and classic papillary carcinoma from papillary hyperplastic nodules can be challenging 10.

Although immunohistochemistry is considered useful in this process, there is controversy over the best markers to distinguish papillary thyroid carcinoma from its mimickers. Various immunostains have been studied to aid in the differential diagnosis of papillary thyroid carcinoma, such as cytokeratin 19 (CK19), Hector Battifora Mesothelial 1 (HBME1), or Fibronectin 1 (FN1) among others 11.

#### 2.1.1. Histological Use of HBME1 and CK19

HBME1 (Hector Battifora Mesothelial-1) is a monoclonal antibody directed against the microvillous surface of mesothelial cells, originally developed as a marker for mesothelioma but subsequently applied to the diagnosis of malignant thyroid conditions. To consider staining positive for HBME1, it must stain the basolateral membrane. Cheung *et al.* 12 reported HBME1 positivity in 70% of patients included in the study with classic papillary thyroid carcinomas and 45% with the follicular variant of papillary thyroid carcinomas. Conversely, HBME1 expression was not observed in 40% of nodular hyperplasia cases and 35% of follicular adenomas. It's worth mentioning that HBME1 expression may be present in focal areas of Hashimoto's thyroiditis, and cells in these areas may exhibit nuclear features of papillary thyroid carcinoma, so HBME1 positivity per se should not be equated with a diagnosis of papillary thyroid carcinoma in this setting 13. In summary, HBME-1 serves as a valuable tool in the diagnostic workup of PTC, aiding in the differentiation of malignant from benign thyroid lesions but is not entirely specific and can be expressed in other types of thyroid carcinomas, such as follicular carcinoma and, less commonly, in medullary thyroid carcinoma.

The diagnostic utility of CK19 in papillary thyroid carcinoma has been controversial. Some studies have observed negative staining of CK19 in benign thyroid lesions and high frequencies of CK19 expression in papillary thyroid carcinoma. The main utility of CK19 relies in its high sensitivity for papillary thyroid carcinoma, so negative staining for CK19 is a strong indication against papillary thyroid carcinoma. FN1 has been proposed as a useful marker for the diagnosis of papillary thyroid carcinoma; however, several studies have observed that the low sensitivity of this staining significantly impairs the utility of FN1 in a diagnostic setting 14,15. Therefore, the utility of HBME1 has been demonstrated, and it is suggested that the combination of HBME1 and CK19 achieves high sensitivity and specificity in the diagnosis of papillary thyroid carcinoma 16.

### 2.2. Serological Biomarkers

In the case of epithelial lineage thyroid tumors, one of the first tissue-specific biomarkers used was thyroglobulin (TG). TG is a glycoprotein produced by the follicular cells of the thyroid gland and is a key component in the synthesis of thyroid hormones.

In patients with papillary thyroid cancer, serum levels of TG may be elevated due to the excessive production of this protein by cancer cells. Therefore, measuring serum thyroglobulin levels can be useful as a marker of residual or recurrent disease after initial treatment, such as thyroidectomy or radioactive iodine ablation therapy 17. Although TG is a useful marker in monitoring papillary thyroid cancer, there are some limitations to its use. For example, patients who still have residual thyroid tissue may have elevated levels of thyroglobulin, which complicates the interpretation of results. Additionally, patients who are under suppressive therapy with thyroid hormone may have lower levels of thyroglobulin, which can affect the sensitivity of the marker 18.

### 2.3. Genetic Biomarkers

Advances in the understanding of genomic alterations in thyroid cancer have transformed clinical practice. Fine-needle aspiration cytology provides an accurate diagnosis in approximately 80% of cases, while the remaining 20% yield indeterminate results. Previously, nodules with indeterminate cytology often required diagnostic surgery, which identified cancer in about one-third of cases. Today, molecular tests with broad panels of DNA/RNA or RNA/NGS markers have been validated and offer high sensitivity and specificity for detecting follicular cell-derived thyroid cancers, as well as parathyroid nodules that may resemble follicular nodules. The introduction of these molecular tests has significantly reduced the need for diagnostic surgeries in patients with thyroid nodules showing indeterminate cytology.

The genetic alterations of papillary thyroid cancer are related to the mitogen-activated protein kinase (MAPK) pathway, which promotes cell division. These tumors may exhibit various genetic anomalies such as activating mutations (BRAF, RAS) and rearrangements (RET and NTRK1) that lead to sequential activation of MAPK. Additional drivers include rearrangements of anaplastic lymphoma kinase (ALK), mutations in EIF1AX, and mutations in the promoter region of the telomerase reverse transcriptase (TERT) gene. The 9% of these tumors may present combined mutations in TERT and BRAF or RAS 19. These exhibit a more aggressive behavior than papillary thyroid cancers carrying a single mutation. RAS mutations are considered weak as they are common in benign thyroid neoplasms and other cancers, whereas RET, NTRK, and ALK rearrangements, along with mutations in BRAF, EIF1AX, and the TERT promoter, are termed strong and are often associated with malignant tumors. Identifying these strong drivers in thyroid nodules suggests a high risk of malignancy and is employed in DNA analyses by various commercial laboratories 20.

#### 2.3.1. Rearrangements of RET and NTRK

Papillary thyroid cancers are associated with rearrangements in three different transmembrane tyrosine kinase genes: RET, NTRK1, and NTRK3. These rearrangements produce chimeric proteins with constitutive tyrosine kinase activity that contributes to the development of the malignant phenotype. The resulting chimeric genes from RET rearrangements are known as RET/PTC, while those from NTRK1 rearrangements are termed TRK. RET encodes a neurotrophic factor receptor, and NTRK1 and NTRK3 encode nerve growth factor receptors, none of which are expressed in normal thyroid cells. Somatic rearrangements of RET or NTRK result in increased tyrosine kinase activity, activating growth pathways and potentially causing papillary cancer and leading to constitutively unregulated activation of TRK kinases and downstream pathways, contributing to tumor development and progression. The frequency of these rearrangements in papillary thyroid cancer varies. In adults, around 40% of sporadic papillary cancer cases have these rearrangements, with RET being the most common. In children, the incidence of RET rearrangements is higher, reaching approximately 60%. This percentage increases significantly in pediatric papillary cancers exposed to radiation 21,22.

In this sense we should highlight that RET gene rearrangement, particularly RET/PTC, is highly specific for papillary thyroid carcinoma and is associated with the characteristic nuclear features seen in this type of cancer 23. As reported before, diverse studies have shown that RET/PTC rearrangements are more common in radiation-associated thyroid tumors, and experimental evidence suggests that exposure to ionizing radiation can induce RET/PTC rearrangements in human thyroid cells 24,25.

The identification of RET and NTRK rearrangements in PTC is crucial for understanding the molecular mechanisms underlying the disease and for developing targeted therapies. The recent approval of anti-TRK agents like larotrectinib and entrectinib underscores the significance of identifying NTRK rearrangements as actionable targets in cancer therapy. Furthermore, genomic-based targeted therapies have been developed for NTRK and RET gene fusion-positive DTCs, emphasizing the importance of molecular profiling in guiding treatment decisions for patients with thyroid cancer.

#### 2.3.2 BRAF Mutations

BRAF mutations are known to play a significant role in papillary thyroid cancer (PTC), with the BRAF V600E mutation being the most common genetic alteration in this type of cancer, occurring in about 45% of sporadic PTC cases in Western countries and up to 90% in Korea 26. It should be noted that the presence of BRAF mutations has been associated with high-risk clinicopathological characteristics like tumor size, extrathyroidal invasion, lymph node metastasis, and higher TNM stage. In this sense it has been linked with tumor recurrence, and reduced sensitivity to radioiodine therapy in adult patients with PTC 27,28. We should remark the importance of BRAF mutations providing opportunities for targeted drug therapy in thyroid cancer. For instance, the BRAFV600E mutation, found in 50% of PTC cases, offers a potential target for therapy in both PTC and anaplastic thyroid cancers 29,30. However, the correlation between BRAF mutation and clinicopathological parameters may vary among different variants of PTC, with the mutation being significantly more common in conventional PTC compared to the follicular variant 31. In conclusion, BRAF mutations, particularly the V600E mutation, are crucial in the pathogenesis of PTC, influencing tumor characteristics, treatment response, and providing avenues for targeted therapies. Understanding the role of BRAF mutations in PTC is essential for improving diagnostic accuracy, treatment strategies, and patient outcomes in thyroid cancer management.

#### 2.3.3 RAS Mutations

On the other hand, mutations in RAS genes are less common compared to BRAF gene mutations and have been reported in the follicular variant of papillary thyroid cancers. Studies have shown distinct patterns of RAS mutations in different histological subtypes of PTC. For instance, RAS mutations were observed in 36% of the encapsulated group compared to 10% in infiltrative tumors, indicating a potential association with specific PTC subtypes 32. Furthermore, RAS mutations have been linked to tumor dedifferentiation and are more prevalent in anaplastic (undifferentiated) thyroid carcinomas, suggesting a role in disease progression and aggressiveness 33.

Within these mutations, NRAS mutations are more common than HRAS mutations. RAS gene mutation acts by activating cellular signaling pathways that promote cell proliferation, survival, and invasion, thereby contributing to tumor growth. Several studies have suggested that the presence of the RAS mutation in papillary thyroid cancer may be associated with more aggressive clinical features and a worse prognosis. This includes a higher likelihood of recurrence and a lower disease-free survival rate compared to patients without this mutation 34.

In conclusion, RAS mutations play a significant role in the molecular pathogenesis of papillary thyroid cancer, influencing tumor behavior, dedifferentiation, and potentially serving as markers for specific histological subtypes. Understanding the implications of RAS mutations in thyroid cancer is crucial for advancing diagnostic and therapeutic strategies in the management of this disease.

#### 2.3.4. TERT Mutations

Mutations in the TERT gene (telomerase reverse transcriptase) are genetic alterations that have been associated with various types of cancer, including thyroid cancer. These mutations, which can occur in different regions of the genome, including the TERT gene promoter, may increase telomerase activity, an enzyme that helps maintain the length of telomeres. Telomerase activation can lead to cellular immortalization and promote uncontrolled cell proliferation, contributing to cancer development and progression 35.

In a study that included 332 cases of papillary thyroid cancer, the presence of the TERT mutation was identified as an independent indicator of persistent disease in well-differentiated thyroid cancer. These mutations have been identified in 7-22% of cases of papillary thyroid cancer, although they are more common in aggressive variants such as anaplastic cancer and have been associated with a worse prognosis 36.

Furthermore, TERT mutations have been shown to enhance the prognostic effects of coexisting BRAF or RAS mutations in differentiated thyroid cancer patients, strengthening risk prediction models and providing valuable insights into the clinical management of thyroid malignancies 37. The association of TERT promoter mutations with distant metastases in PTC underscores their role in predicting aggressive behavior and aiding in risk stratification for patients with this type of tumor 38. In conclusion, TERT mutations play a crucial role in the pathogenesis of papillary thyroid cancer, influencing tumor aggressiveness, recurrence, and patient prognosis. Understanding the implications of TERT promoter mutations in thyroid cancer is essential for personalized treatment strategies, risk assessment, and improving clinical outcomes in individuals with PTC.

#### 2.3.5. NTRK Fusion

NTRK gene fusions (Neurotrophic Tyrosine Receptor Kinase) can lead to a hybrid NTRK protein with abnormal signaling activity. This abnormal activation can contribute to the development and progression of cancer in certain types of tumors. These fusions are rare but have been identified in different histological variants of thyroid carcinoma 39. NTRK fusion, like other genetic alterations, is more commonly associated with an earlier age of diagnosis, has been linked to a follicular growth pattern, and may be more associated with locoregional disease and distant metastasis.

Furthermore, NTRK gene fusions have been found to be mutually exclusive with other primary oncogenic drivers, such as RAS family genes and BRAF mutations, in thyroid carcinoma 40. This exclusivity suggests that NTRK fusions may represent a unique molecular subtype of thyroid cancer with specific biological implications. Additionally, NTRK fusion-positive PTCs have been associated with increased metastatic capacity and persistent disease, highlighting the clinical significance of these genetic alterations in predicting patient outcomes and guiding treatment decisions 41.

In terms of therapeutic implications, NTRK inhibitors have shown promise in the treatment of NTRK fusion-positive cancers, including thyroid carcinomas. Targeted therapies, such as larotrectinib, have demonstrated efficacy in patients with NTRK fusion-positive high-grade cancers, leading to remission and improved clinical outcomes. The identification of NTRK gene fusions in thyroid cancers has opened up new avenues for precision medicine approaches, offering potential targeted treatment options for patients with this molecular subtype of PTC 42.

In conclusion, NTRK gene fusions represent a distinct molecular alteration in papillary thyroid cancer, with implications for tumor biology, clinical behavior, and therapeutic interventions. Understanding the prevalence, clinical significance, and therapeutic implications of NTRK fusions in PTC is essential for advancing personalized treatment strategies and improving outcomes for patients with this type of thyroid cancer.

### 2.4. Circulating Tumor Cells, Circulating Tumor DNA, and MicroRNAs

Liquid biopsy, as a non-invasive and accessible method for detecting tumor cells or tumor-derived products in bodily fluids, has seen significant development in recent years. It can identify genetic material generated by tumors, such as circulating tumor cells (CTCs), circulating tumor DNA (ctDNA), exosomes, circulating free DNA (cf-DNA), and circulating free RNA (cf-RNA), present in peripheral blood, saliva, urine, and cerebrospinal fluid. CTCs, ctDNA, and exosomes are more stable and specific than cf-RNA and cf-DNA, making them stand out in the clinical application of liquid biopsy 43.

#### 2.4.1 Circulating Tumor Cells (CTCs)

Circulating tumor cells (CTCs) are cancer cells that detach from the primary tumor and enter the bloodstream or lymphatic circulation. They can be quantified using techniques such as flow cytometry, immunomagnetism, fluorescence microscopy, quantitative PCR, and microfluidics-based techniques. These cells are responsible for cancer spread, which can lead to metastasis in other organs.

In 1998, Ringel *et al.* confirmed the presence of CTCs in the blood of patients with thyroid cancer and local recurrence or distant metastasis 44. Since then, interest in the role of CTCs in this type of cancer has grown considerably. CTC detection could offer new opportunities for cancer diagnosis, especially in early stages, as it can anticipate metastasis presence and provide accurate prognostic data.

Moreover, it has been observed that patients with a higher number of CTCs present more aggressive tumor characteristics, such as larger tumors, multifocality, and vascular invasion, resulting in overall poorer survival and worse treatment response. This suggests their utility in risk stratification and clinical decision-making. However, it is important to note that most studies are retrospective and have limitations, such as small sample sizes and limited follow-up times 45.

Several studies have demonstrated a correlation between the number of CTCs and tumor stage, as well as with the response to radioactive iodine (131I) treatment. For instance, Xu *et al.* 46 used the CellSearch system to detect CTC numbers in the peripheral blood of metastatic thyroid cancer patients and found that patients with CTCs ≥ 5 had a median overall survival of 51.5 months, whereas that of patients with CTCs ≥ 5 was 13 months. Qiu *et al.* detected CTCs in patients with differentiated thyroid carcinoma and found that those with ≥7 CTCs were related to poor response to 131I therapy, suggesting a poor prognosis 46, 47. Winkens *et al.* identified CTCs in patients with differentiated thyroid cancer before and after radioactive iodine (RAI) treatment and found that a decrease in CTC quantity in the early stages of RAI therapy could identify treatment-sensitive patients 48.

In conclusion, CTCs represent a promising avenue for improving the diagnosis, monitoring, and prognostication of papillary thyroid cancer. The assessment of CTCs in PTC patients holds significant potential for enhancing risk stratification, treatment selection, and overall clinical management of thyroid cancer, ultimately contributing to improved patient care and outcomes.

#### 2.4.2. Circulating Tumor DNA (ctDNA)

Circulating tumor DNA (ctDNA) is a fraction of cell-free DNA that represents genetic changes present in tumors and has been highlighted as a promising biomarker for cancer diagnosis and monitoring. Its detection in bodily fluids requires highly sensitive techniques such as quantitative real-time PCR and next-generation sequencing (NGS). Although NGS offers advantages in sequence data generation, its clinical applicability may be limited. However, ctDNA's short half-life allows real-time monitoring of cancer progression, making it a valuable tool for understanding tumor heterogeneity and improving disease management 49.

So far, the role of ctDNA in thyroid cancer has been evaluated in few research studies, so its role is not yet clearly defined; however, there is some evidence of its potential applications. For example, Chuang *et al.* 50 discovered that 21% of patients with papillary thyroid carcinoma had detectable BRAF mutations in their circulating DNA, while benign patients did not show such mutation. About 44.07% of thyroid tumors were positive for BRAF-V600E ctDNA, suggesting its utility in thyroid nodule detection. Lan *et al.* 51 observed a higher ctDNA detection rate in papillary thyroid cancer with distant metastasis compared to those without metastasis. Allin *et al.* 52 detected ctDNA in 67% of patients with a sensitivity of 76%, suggesting that ctDNA could be a useful biomarker in therapeutic monitoring of thyroid cancer due to its rapid variation in response to disease status compared to traditional markers.

The ctDNA methylation has been associated with specific tumor alterations and is considered a promising biomarker in various cancer types, including thyroid cancer. Hypermethylation of CpG island promoters has been highlighted as a crucial component in the inactivation of tumor suppressor genes in this cancer type. Several studies have shown that hypermethylation of specific genes, such as MGMT, SLC5A8, and SLC26A4, may be useful in the diagnosis and monitoring of thyroid cancer recurrence 53. Serum DNA methylation has emerged as an effective diagnostic tool, with markers such as CALCA, CDH1, TIMP3, DAPK, and RARβ2 showing utility in both initial diagnosis and recurrence detection 54.

The use of ctDNA detection in thyroid cancer patients is currently a topic of debate. Despite its potential as a diagnostic biomarker, its clinical application in this context is still limited. Several studies have encountered challenges in detecting specific mutations, such as BRAFV600E, in ctDNA from thyroid cancer patients, even with highly sensitive techniques. Moreover, there is a lack of clear correlation between mutations found in tumor tissue and those identified in plasma mutational data in comparative studies. These findings indicate that the effectiveness of ctDNA in diagnosing and monitoring thyroid cancer is not yet fully established, suggesting caution in its incorporation into current clinical practices, as indicated by some research 55.

In conclusion circulating tumor DNA emerges as a promising tool for non-invasive cancer monitoring, showing potential in thyroid cancer for detecting mutations and disease monitoring. However, its clinical application is still limited by challenges in mutation detection and correlating ctDNA with tumor tissue. Further research is needed to validate ctDNA's utility in clinical practice for thyroid cancer.

#### 2.4.3. MicroRNAs

MicroRNAs (miRNAs) are a class of small RNA molecules that play an important role in gene expression regulation in cells. These are short RNA fragments of approximately 20 to 22 nucleotides in length. These molecules are crucial in post-transcriptional gene expression regulation. In the context of cancer, miRNAs can influence tumor progression, angiogenesis, invasion, and metastasis, among other processes. Therefore, studying miRNAs in cancer can provide valuable information about tumor biology, as well as offer possible therapeutic opportunities or biomarkers for cancer diagnosis and monitoring. Several studies demonstrate the efficacy of miRNA-based analyses in discriminating high-risk tumor mutations and for detecting metastases 56.

Recent studies have assessed the expression of specific miRNAs in patients with papillary thyroid carcinoma. These miRNAs can serve as useful markers for predicting the likelihood of cancer recurrence after initial treatment. Their early detection in treated patients can help identify those at higher risk of recurrence and allow for more effective and personalized interventions.

Some studies have revealed that various miRNAs can serve as biomarkers for the diagnosis of papillary thyroid cancer, such as miR-223-3p, miR-34-5p, miR182-5p, miR-146b-5p, miR-29a, miR-223-5p, miR-16-2-3p, miR-34a-5p, miR-346, miR-10a-5p, miR-485-3p, miR-4433a-5p, and miR-5189-3p. Additionally, studies have shown that certain miRNAs like miR-31 and miR-21 can help distinguish between different types of thyroid cancer, such as papillary carcinoma and follicular carcinoma. MiR-145 has also been identified as a promising marker for papillary thyroid cancer invasion, while miR-6879-5p and miR-6774-3p show a significant increase in patients with lymph node metastasis 57.

On the other hand, early detection of thyroid cancer recurrence is crucial. Studies have demonstrated that miRNAs play a role in thyroid cancer metastasis and recurrence. Specific concentrations of exosomal miRNAs, such as miR-29a, have been linked to recurrence of papillary thyroid carcinoma. Additionally, miR-146b and miR-222 may serve as recurrence markers in papillary thyroid carcinoma 58.

In papillary thyroid carcinoma, specific miRNAs have been identified as potential markers for cancer diagnosis, prognosis, and recurrence prediction. These miRNAs, including miR-223-3p, miR-34-5p, and others, offer insights into the likelihood of cancer recurrence and can distinguish between thyroid cancer types. Furthermore, miRNAs such as miR-145 and miR-6879-5p are linked to cancer invasion and lymph node metastasis, respectively. Early detection of these miRNAs can facilitate personalized treatment strategies, highlighting their importance in improving patient outcomes in thyroid cancer.

## 3. Thyroid Follicular Carcinoma

Thyroid follicular carcinoma stands as a significant entity among thyroid cancers, representing a subset distinct from the more prevalent papillary thyroid carcinoma (PTC). Characterized by its unique histological features that differentiate it from PTC, follicular carcinoma accounts for approximately 10-15% of all thyroid malignancies. Unlike PTC, follicular carcinoma often presents a more aggressive behavior, particularly in its ability to invade blood vessels and metastasize to distant organs 59.

The prognosis for patients with follicular thyroid carcinoma is generally good, but it is characterized by its tendency to invade blood vessels and spread to distant locations such as bones and lungs through the bloodstream, with distant metastasis rates reported between 6-20%. Distant metastasis can either be an initial sign of FTC or develop post-initial treatment. Post-total thyroidectomy distant metastasis incidence ranges from 7% to 23%, while initial presentation with distant metastasis occurs in about 1-9% of cases so the prognosis is related to distant metastatic disease 60.

Advancements in molecular diagnostics have begun to delineate a more precise therapeutic landscape for follicular carcinoma. For instance, targeted therapies against specific genetic alterations are being explored, offering hope for more personalized and effective treatment approaches. Despite these advances, the challenge remains to integrate these biomarkers into clinical practice fully, ensuring that patients with follicular thyroid carcinoma receive the most informed and tailored care possible.

In conclusion, while follicular thyroid carcinoma presents a generally favorable outcome, its management is nuanced by the tumor's potential for aggressive behavior and distant metastasis. The ongoing identification and integration of specific biomarkers are crucial for enhancing diagnostic accuracy, enabling risk stratification, and guiding therapeutic decisions, ultimately leading to improved patient outcomes in this diverse group of thyroid cancers.

### 3.1. Histological Biomarkers

The follicular-patterned thyroid lesions (FPTLs) include hyperplastic nodules (HN), follicular adenoma (FA), non-invasive follicular neoplasm with papillary-like nuclear features (NIFTP), follicular carcinoma (FC), and the follicular variant of papillary carcinoma (FVPTC). Sometimes the pathologists cannot accurately separate these lesions from each other on a histological basis.

At times, the histological distinction between well-differentiated thyroid carcinomas with follicular patterns (FVPTC and FC) and benign FPTLs (HN and FA) can be complicated. Therefore, obtaining information about the differences in the expression pattern of some immunostains in FPTLs can help resolve this issue 61.

These immunostains include HBME-1, Galectin-3, cytokeratin 19 (CK19), and CD56. The expression of HBME-1, Galectin-3, and CK19 is absent in normal thyroid follicular cells (2). HBME-1 is a marker of mesothelial cells 62 found in their microvilli. Galectin-3 is a lectin family protein that binds to cell surface glycoprotein and participates in cell signaling, cell-cell adhesion, cell-matrix interaction, metastasis, and apoptosis. Galectin-3 is absent in normal thyroid follicular cells 63. CK19 is a small cytokeratin that participates in the organization of myofibrils within simple and complex epithelial cells. CD56 is a neural cell adhesion molecule encoded by a gene located on chromosome 11q23. The reduction or loss of CD56 expression correlates with thyroid cancer 64.

Some previous studies suggested a diagnostic utility of HBME-1, Galectin-3, CK19, and CD56 to separate malignant PTC from benign FPTL (such as FA and HN), but none of these immunostains is individually reliable to establish a firm diagnosis 64-66. Their main limitation in diagnosing malignant FPTL is their inconsistent expression in some benign FPTL. In this sense Nechifor-Boila, *et al.* examined the expression of HBME-1, Galectin-3, Cytokeratin-19, and CD56 in a series of PTC and FTUMP 67. CD56, whose expression is reduced or absent in thyroid carcinomas, was the most sensitive marker (81.8%), showing a "malignant" profile in most of the PTC. It was followed by HBME-1 (63.6% sensitivity). Cytokeratin-19 and Galectin-3 were the least sensitive antibodies (45.6%) but the most specific (100%). In FTUMP, Cytokeratin-19, Galectin-3, HBME-1, and CD56 were negatively stained in most cases 67.

Although some studies have been conducted on the expression of individual markers, especially in PTC, our knowledge about the expression profile of the four markers in various benign FPTLs (HN and FA) and malignant FPTLs (FVPTC and FC) is limited. Understanding the expression patterns of these lesions will provide valuable information for their diagnosis.

In summary, FPTLs remain a diagnostic dilemma for pathologists and clinicians. The discrepancy in their diagnostic utility among different studies is due to the use of different cutoff values and the lack of consensus on the staining pattern of these proteins (cytoplasmic ± membranous). HBME-1, Galectin-3, and CK19 are frequently expressed in malignant FPTLs, but they are also inconsistently expressed in benign FPTLs. Therefore, it is prudent to use all four markers in combination to help differentiate carcinomas from HNs and FA.

Additionally, the evaluation of routine hematoxylin/eosin-stained sections will continue to be the gold standard for the diagnosis of FPTLs until future studies can find more reliable markers.

### 3.2. Serological Biomarkers

Thyroglobulin (TG) is a protein specifically produced by the follicular cells of the thyroid and is a key component in the synthesis of triiodothyronine (T3) and thyroxine (T4). The detection of thyroglobulin in the biopsy determines a thyroid origin of the tissue.

In the case of follicular thyroid cancer, TG can be a useful marker for disease monitoring. Serum TG levels may be elevated due to the excessive production of this protein by tumor cells. Measuring serum TG levels is very useful in monitoring and detecting residual or recurrent disease after initial radical treatment such as thyroidectomy or radioactive iodine ablation therapy. Currently, serum thyroglobulin is measured using immunometric assays "sandwich" of two antibodies with isotopic labeling method (immunoradiometric assay, IRMA) or non-isotopic (usually immunochemilumin escent assay, ICMA). These immunometric assays are faster and more sensitive, allowing detection of thyroglobulin levels from 0.1 to 1 ng/ml 68.

However, there are some limitations in the use of TG as a marker. For example, in those patients with residual thyroid tissue that continue to produce TG, it may be difficult to interpret elevated TG levels. Therefore, TG can be a useful marker in the monitoring of follicular thyroid cancer, but it is important to know its limitations and consider other clinical factors when interpreting the results.

### 3.3. Genetic Biomarkers

Although most FTCs are sporadic, approximately 5% of cases are associated with a genetic predisposition. Several genetic markers have been identified that may be involved in the development and progression of FTC.

#### 3.3.1. RAS Mutations

Point mutations in the RAS oncogene, including HRAS, KRAS, and NRAS, are observed in approximately 40% of FTCs 69. RAS oncogene mutations occur more frequently in follicular thyroid cancers than in follicular adenomas. However, RAS mutations are not specific to FTC; they are also found in a small proportion of papillary thyroid cancers, particularly in the follicular variant of papillary thyroid cancer 69,70. RAS mutations in FTC can activate the MAPK signaling pathway, which may promote cell proliferation and survival and appear to be associated with more aggressive tumors and consequently higher mortality 70.

#### 3.3.2 PAX8 PPAR-gamma-1 Rearrangement

PAX8-PPAR gamma 1 is a genetic rearrangement observed in 10% of adenomas and 41% of follicular cancers. This fusion protein can help distinguish between follicular adenoma and carcinoma 71. It is believed that PAX8-PPAR gamma 1 induces a negative effect on the activation of thiazolidinediones by PPAR gamma, leading to the loss of growth inhibitory controls 72,73.

In conclusion the genetic rearrangements involving the PAX8 and PPARG genes are characteristic of thyroid follicular cancer and are associated with a more favorable prognosis and greater survival.

Thyroid follicular cancer presents either the RAS mutation or the PAX8-PPAR gamma 1 rearrangement, but not both.

#### 3.3.3. TERT Mutations

Mutations in the TERT (telomerase reverse transcriptase) gene are an important genetic biomarker in FTC. Telomerase is an enzyme that helps maintain the length of telomeres, which are the protective structures at the ends of chromosomes. Mutations in the TERT gene can activate telomerase and promote uncontrolled cell proliferation, which can contribute to the development and progression of cancer 74. TERT promoter mutation has been detected in 17% of FTC, a frequency that is higher than that seen in PTC (9%) 75. In this sense these mutations are also more commonly associated with aggressive tumor behaviors and poor clinical outcomes, including tumor recurrence and patient mortality.

#### 3.3.4. PTEN Mutations

PTEN is a tumor suppressor gene that regulates the PI3K/AKT/mTOR signaling pathway, which is involved in cell proliferation and survival. Mutation of this gene is associated with a familial neoplastic syndrome (Cowden syndrome) characterized by the predominant formation of multiple hamartomas and a predisposition to the development of thyroid cancer, predominantly papillary, although FTC may be overrepresented compared to the general population and is associated with a higher risk of recurrence and metastasis in FTC. 76.

#### 3.3.4. TSHR Mutations

Mutations in the TSHR gene (thyroid-stimulating hormone receptor) are an important genetic biomarker in follicular thyroid cancer. TSHR is a receptor on the surface of thyroid cells that responds to thyroid-stimulating hormone (TSH) and plays a crucial role in regulating thyroid function. Mutations in the TSHR gene can alter receptor function and contribute to the development and progression of follicular thyroid cancer. They are found in 20%-82% of hyperfunctioning nodules, hyperfunctioning FTC, and PTC 77. *In vitro* studies suggest a possible role for EGF as a modulator of angiogenesis in thyrocytes lacking TSHR 78. Mutations in the TSHR gene have been associated with a higher risk of recurrence and metastasis in FTC 79.

#### 3.3.5. EIF1AX Mutations

Mutations in the EIF1AX gene (eukaryotic translation initiation factor 1A, X-linked) are an important genetic biomarker in follicular thyroid cancer. EIF1AX is a translation initiation factor that plays a crucial role in the initiation of protein synthesis in cells. EIF1AX mutations occur in benign and malignant follicular thyroid neoplasms 80. Mutations in the EIF1AX gene can alter the function of this factor and contribute to the development and progression of follicular thyroid cancer. It also should be noted that these mutations have been associated with a more favorable prognosis and greater survival in follicular thyroid cancer 80.

### 3.4. MicroRNAs

Numerous studies have examined the use of miRNAs as potential biomarkers for the diagnosis, prognosis, and therapeutic targets of human TC; they need to be further validated using a sizable cohort of samples. There is significant evidence that miRNAs are essential for regulating the development and progression of malignant tumors 81.

Unlike papillary thyroid cancer, follicular adenomas and carcinomas share the same tumorigenic pathway. Many studies have shown that the mRNA/miRNA expression profiles of follicular neoplasms differ from those of papillary carcinoma; however, molecular signatures that clearly distinguish between follicular adenoma and follicular carcinoma need further validation in large studies 82. In a search for biomarkers to distinguish follicular thyroid cancer and PTC, dysregulated miRNA expression is well documented in follicular neoplasms.

Samsonov *et al.* found that miRNA-21 and miRNA-181a-5p (exosomal miRNA) can differentiate between the two types of thyroid cancer with high sensitivity (100%) and specificity (77%) 83.

The expression of certain miRNAs such as miR-197 and miR-346 is more frequently observed in follicular carcinoma than in follicular adenoma 84,85. In this meta-analysis 84, miR-637, miR-181c-3p, miR-206, and miR-7-5p were identified as potential biomarkers of FTC, with miR-7-5p being particularly significant as a biomarker to distinguish between benign and malignant thyroid tissue.

The distribution of miRNAs in human tumors exhibits traits associated with a cancer diagnosis, prognosis, staging, and therapeutic response. miR-19a is one of the oncomiRs whose impact on TC has been studied. When this member of the miR-17-92 cluster is overexpressed, the cells it is associated with become more invasive and less well-defined in TC tissues. This miRNA was over-expressed artificially in well-differentiated FTC cells, and the result was increased cell proliferation and a change in the expression profile of genes involved in thyroid cell differentiation and aggressiveness, such as TSH receptors and thyroglobulin 86.

MiRNAs are known for their ability to influence various signaling pathways, either by stimulating or inhibiting them. They are also highly stable molecules, resistant to RNases, and can be found in numerous cellular compartments and fluids. These characteristics make miRNAs a promising avenue for cancer research, particularly in the areas of diagnosis, treatment, and monitoring. In thyroid cancer (TC), miR-221, miR-146, and miR-222 have shown particular promise. MiR-221-3p has demonstrated 100% specificity in certain populations, while miR-146b has shown a sensitivity of 94.3% 87. The altered expression of these miRNAs (miR-146b and miR-221) has been associated with a worse prognosis in FTC.

### 3.5 Clinical Applications in Differentiated Thyroid Cancer

Biomarkers play an essential role in thyroid cancer by facilitating early diagnosis, stratifying risk, predicting treatment response, and improving long-term patient monitoring. Their integration into clinical practice can significantly contribute to more personalized and effective patient care. Liquid biopsies offer a less invasive alternative to tumor biopsies, allowing for repetitive sampling and reducing financial costs and potential complications. Results from liquid biopsies can guide disease diagnosis and management. Thyroid cancer, being one of the most common endocrine tumors, has been the subject of numerous studies related to liquid biopsies 88.

In terms of diagnosis, biomarkers can aid in differentiating between benign and malignant thyroid nodules, facilitating clinical decision-making regarding the need for biopsies or surgical interventions. The histological distinction between well-differentiated thyroid carcinomas with follicular patterns and benign forms can be complicated. Therefore, obtaining information about the differences in the expression pattern of certain immunostains can help resolve this problem. These immunostains include HBME-1, Galectin-3, cytokeratin 19, and CD56 89.

Differentiated thyroid cancer, especially papillary thyroid carcinoma, are generally associated with a better prognosis compared to other thyroid cancer subtypes such as medullary or anaplastic. However, certain genetic mutations have been linked to a worse tumor prognosis, with BRAF V600E mutation being the most common 90. However, other mutations such as those in the EIF1AX gene have been associated with a favorable prognosis. Additionally, quantification of circulating tumor cells (CTCs) and certain miRNAs has been implicated in biological processes favoring tumor growth and spread, aiding in patient stratification and treatment planning 91.

On the other hand, some biomarkers can predict patients' response to certain treatments, such as radioactive iodine therapy or targeted therapies. This allows for a more precise selection of therapy and can improve treatment outcomes. Some studies have observed that patients with a higher number of CTCs associate with a poorer treatment response, conferring a more precarious prognosis 92. A better understanding of the molecular biology of thyroid cancer allows for the identification of new therapeutic targets, which may lead to the development of more specific targeted treatments against the tumor. BRAF inhibitors, such as vemurafenib, encorafenib, or dabrafenib, have shown benefit in clinical trials in patients with thyroid cancer carrying the BRAFV600E mutation 93. However, it is important to note that not all patients respond in the same way to these treatments, as some may experience resistance. MEK inhibitors, such as trametinib, cobimetinib, or binimetinib, are medications that have been investigated in the treatment of thyroid cancer, often combined with BRAF inhibitors as they block key points in the MAPK signaling pathway; this combination can improve efficacy and reduce treatment resistance 93. NTRK inhibitors, such as larotrectinib and entrectinib, are being investigated in the treatment of thyroid cancer, including the papillary variant. Although these fusions are less common compared to other types of cancer, their presence has been documented in a small but significant proportion of thyroid cancer cases. Waquespack *et al.* 94 observed a benefit when using larotrectinib in patients with advanced non-medullary thyroid carcinoma and NTRK fusion.

Regarding disease monitoring in differentiated thyroid cancer, serum thyroglobulin determination is an important tool in patient follow-up after initial treatment. After thyroidectomy and radioactive iodine ablation therapy, it is expected that serum thyroglobulin levels will decrease. If thyroglobulin levels are undetectable or very low, this suggests a favorable treatment response and biochemical remission. However, if thyroglobulin levels increase or remain elevated, it may indicate the presence of residual thyroid tissue or recurrence of the disease 95.

## 4. Medullary Thyroid Carcinoma

Medullary thyroid carcinoma (MTC) is a neuroendocrine malignant tumor that originates from the parafollicular cells or C cells of the thyroid, which embryologically derive from the neural crest. Its frequency is not well established, but it accounts for approximately 2-5% of all thyroid cancers and 0.4-1.4% of thyroid nodules 96. Data from different registries show an incidence of MTC in Europe of 1500-2000 new cases per year and a prevalence of 0.10 to 0.22 per 1,000,000 inhabitants 97. It should be noted that 75% of MTCs are sporadic, with the rest being hereditary. The latter are part of multiple endocrine neoplasia type 2 (MEN2). MEN2 is a syndrome with autosomal dominant inheritance due to a germline activating mutation of the RET oncogene. 98.

Medullary thyroid carcinoma (MTC) is a rare neuroendocrine malignant tumor derived from the parafollicular cells or thyroid C cells. At the time of diagnosis, over 50 % of patients have lymph node metastases, and 10 % have distant metastases. Prognosis is largely dependent on tumor stage and, therefore, early diagnosis is essential.

### 4.1. Histological Biomarkers

Thyroid tumor pathology is diverse 99 and often poses a significant diagnostic challenge. The most common thyroid carcinoma is differentiated carcinoma, which has a rather favorable prognosis when managed with surgery and iodine therapy. 100 Medullary thyroid carcinoma (MTC) is an uncommon condition (3-10% of thyroid neoplasms) arising from parafollicular C cells and may be sporadic or familial in nature. Familial MTC, which is associated with a characteristic phenotype, is the predominant form 101.

MTC is an uncommon tumor and thus difficult to study because of the limited number of patients and series 102. In addition, from the histological viewpoint, MTC may show a great variability in its cytoarchitectural characteristics, which makes it difficult to recognize and may require the use of immunochemistry techniques for diagnosis. There is also no agreement regarding the classification of these tumors into different cytoarchitectural patterns.

However, the immunohistochemical profile of these tumors is poorly known because of their uncommon occurrence and the heterogeneity of the methods used. Thus, assessment of results obtained with immunohistochemical staining is controversial, because not all authors agree as to how positivity should be quantified and there may be changes in the sensitivity of the different antisera used 103.

MTCs have a solid growth pattern and plasmacytoid cells, with frequent presence of amyloid, C-cell hyperplasia, multicentricity, and neovascularization. Immunoreactivity is high for calcitonin, CEA, and bcl-2, low for Ki-67, and intermediate for p53 and C-erb-B2. A comparison of sporadic and familial cases shows that C-cell hyperplasia and multicentricity more commonly occur in familial MTC, but are not exclusive to this tumor, while necrotic foci, bleeding, vascular invasion, and neovascularization are more common in sporadic MTC. Neither histopathological nor immunohistochemical criteria are helpful in differentiating the familial and sporadic forms of MTC 104.

### 4.2. Serological Biomarkers

In most cases, the reason for consultation is usually the appearance of a palpable thyroid nodule or cervical adenopathy. It can also manifest with symptoms of dysphagia or dysphonia or present as an incidental thyroid nodule discovered in an imaging test.

Parafollicular cells secrete calcitonin, which is the main biochemical marker for the diagnosis and follow-up of these patients. Generally, this excess of calcitonin does not produce symptoms, but sometimes it is associated with hypersecretion of calcitonin gene-related peptide and can present with diarrhea or vasomotor flushing.

In addition, sometimes MTC can secrete other peptides such as serotonin (which can produce a carcinoid syndrome), corticotropin-releasing hormone CRH, adrenocorticotropic hormone ACTH (causing Cushing's syndrome), histamine, neurotensin, somatostatin, and various gastrointestinal hormones such as chromogranin A. It also produces carcinoembryonic antigen (CEA) which, although less sensitive and specific than calcitonin, is also useful in the follow-up of MTC. 105

The use of calcitonin as a screening tool in patients with nodular thyroid disease is a controversial topic. In favor, it is a highly sensitive marker that could allow early diagnosis. However, the cut-off points are unclear, and it is a tumor of low prevalence. A recent systematic review of 72,368 patients from 16 studies found that only 0.32% of patients with thyroid nodules were diagnosed with MTC 106.

On the other hand, calcitonin elevation is highly sensitive for MTC, but not very specific. Despite the use of high-specificity immunoradiometric assays, calcitonin elevations can be found for technical reasons, other pathological conditions, various drug treatments, and alcohol and tobacco consumption, although levels are generally lower in these cases 107.

Regarding the thyroid, almost half of C-cell hyperplasia cases are associated with a slight elevation of basal and stimulated calcitonin, and papillary and follicular thyroid carcinomas can also be associated with C-cell hyperplasia and slightly elevated calcitonin levels. In addition, there are conflicting data regarding autoimmune thyroiditis, and cases of elevated calcitonin in multinodular goiter have been described.

It should also be noted that calcitonin levels should be interpreted according to age and gender. Men have twice as many C cells as women, with normal levels in men being less than 10 pg/ml and in women less than 5 pg/ml 107.

On the other hand, although much less frequent, false negatives can also be found. Rare cases of MTC, generally sporadic, with calcitonin levels within the normal range have been described 106, as well as interference of immunoassays due to storage problems or very high calcitonin levels (Hook effect).

Calcitonin levels above 100 pg/ml are considered to have a positive predictive value (PPV) of 100% for macroscopic MTC, but the difficulty lies in the intermediate values. If levels are between 10 and 100 pg/ml, it may be useful to determine calcitonin after stimulation with pentagastrin or calcium, although some authors have not found superiority in its diagnostic capacity compared to basal calcitonin with the new immunoassays 108.

The pentagastrin test consists of the injection of 0.5 mg/kg of intravenous pentagastrin in 3 minutes with determination of calcitonin at 3, 5, and 10 minutes after injection. The calcium stimulation test is performed with the intravenous injection of 2 mg/kg of calcium in 1 minute and the determination of calcitonin at 2, 5, and 10 minutes. Due to the limited availability of pentagastrin in recent years, calcium stimulation has been used more and more frequently, and a good correlation has been shown between the two tests 109.

However, it is not clear which cut-off points consistently discriminate MTC from other conditions, although generally in other pathologies or circumstances other than MTC, calcitonin does not increase after stimulus tests, or does so minimally. Thus, stimulated calcitonin levels below 100 pg/ml are associated with a very low risk of MTC and levels above 1000 pg/ml would confirm the presence of MTC with certainty 107.

On the other hand, some studies have observed a high correlation between procalcitonin and calcitonin in patients with MTC. Procalcitonin has greater stability and less circadian variability than calcitonin, and some authors propose that it could be a complementary tumor marker, both for diagnosis and for follow-up of these patients. However, the recommended cut-off point is not clear, although most agree on considering 0.1 ng/ml. In addition, it should be noted that its usefulness in hospitalized patients is limited by the presence of intercurrent infections that could elevate it 109.

### 4.3. Genetic Biomarkers

Medullary thyroid carcinoma (MTC) is a neuroendocrine malignant tumor that originates from the parafollicular cells or C cells of the thyroid, which embryologically derive from the neural crest. It should be remarked that 75% of MTCs are sporadic, with the rest being hereditary. The latter are part of multiple endocrine neoplasia type 2 (MEN2). MEN2 is a syndrome with autosomal dominant inheritance due to a germline activating mutation of the RET oncogene. The RET gene is located on chromosome 10, in the 10q11.2 region, consists of 21 exons, and encodes a transmembrane tyrosine kinase receptor. Two subtypes of MEN have been described: MEN 2A and MEN 2B. In both, MTC is generally bilateral and multicentric, and there is a high genotype-phenotype correlation. MEN 2A is the most frequent, accounting for 95% of hereditary MTC cases, and the classic form associates hyperparathyroidism (HPT) and pheochromocytoma with different frequencies. MEN 2B accounts for 5% and is characterized by MTC in 100% of carriers, is more aggressive, and appears in the first years of life. Additionally, they may present with mucosal ganglioneuromas, intestinal ganglioneuromatosis, pheochromocytoma, and a marfanoid habitus. 98, 110, 111.

### 4.4. Circulating Tumor Cells and MicroRNAs

We should highlight that CTC evaluation has been investigated also in MTC with promising results. In 2022, in a cohort of 12 MTC patients out of 164 patients (7.3%), Weng and colleagues 112 evaluated the number of CTCs at diagnosis using the CanPatrol capture technique and found that 58.3% (7/12 MTC patients) were CTC-positive while 5/12 (41.6%) were not. The authors considered a cutoff of six CTCs and found that, in that case, patients had shorter progression-free survival and an increased metastasis rate. The paper by Weng and colleagues strongly validates the use of CTCs as a prognosis biomarker once a clear cutoff is confirmed. In the same year, a study involving 30 MTC patients out of 394 total subjects (7.6%) validated a six-cell cutoff in an independent series. The authors showed that in MTC, overall survival (OS) was found to be significantly shorter (*p* < 0.01) in individuals with more than six CTCs compared to those with six or fewer CTCs 113.

One study from 2018 114 searched for these types of CTCs using an optimized technique in nine MTCs with a serum calcitonin concentration ranging from 48 to 10,600 pg/mL. Calcitonin-positive CTCs were identified in eight out of nine (89%) patients, but the CTC counts did not always align with the TNM staging system. The authors found that one patient classified as pT3N1bM1 did not exhibit detectable calcitonin-positive CTCs, while another subject, classified as pT1N1bMx, had five CTCs 114. Even a correlation between serum calcitonin concentrations and CTC counts was missing 114. On the contrary, a correlation between the number of CTCs and the T stage at initial diagnosis (Pearson r = 0.9837, *p* = 0.0163) was found in a study by Ehlers and colleagues 115. However, neither lymph node nor distant metastasis nor CT levels had an impact on the number of CTCs in that study 115.

The emerging message from these studies is that the search for CTCs in MTC is feasible and represents a specific test. An elevated number (from four to >six) of circulating tumor cells in patients with MTC may serve as a robust biomarker for predicting cancer prognosis independently from the method used for their extraction and characterization and may improve patient outcomes using more personalized and targeted treatments. Nevertheless, more efforts are needed to find a clear cutoff that is able to detect disease recurrence even many years after surgery and particularly in CT-negative cases.

## 5. Anaplastic Thyroid Cancer (ATC)

ATC is characterized by its rapid progression, resistance to conventional therapies, and poor prognosis. This highly aggressive form of thyroid cancer, although accounting for less than 5% of all thyroid cancer cases, shows a higher incidence in older adults, usually over the age of 60, with a slight female predominance, mirroring trends observed in other thyroid malignancies. The median survival for patients diagnosed with ATC is starkly low, approximately 6 months from the time of diagnosis, reflecting the aggressive nature of this disease. Survival rates have shown little improvement over the years, underscoring the urgent need for novel diagnostic and therapeutic approaches. The poor prognosis is largely attributable to the rapid growth and early metastatic spread of ATC, often rendering surgical options infeasible and limiting the efficacy of radiotherapy and chemotherapy 116.

In this context, the exploration and integration of biomarkers in ATC offer a promising avenue for enhancing our understanding of the disease, improving diagnostic precision, and identifying potential targets for innovative treatments. This segment of the article explores the critical role of histological, serological, genetic biomarkers, as well as circulating tumor cells (CTCs) and microRNAs, in revolutionizing the management of ATC. These biomarkers could represent a new approach for personalized medicine, aiming to improve survival and quality of life for this subgroup of patients.

### 5.1. Histological Biomarkers

Immunohistochemistry is a vital tool for identifying histological markers in anaplastic thyroid cancer (ATC). These markers provide crucial information for accurate diagnosis and the design of treatment strategies in this highly aggressive disease.

#### 5.1.1 PDL-1

The interaction between PD-1 (T cell receptor) and PDL1 (programmed death ligand) is crucial in regulating the immune response. In anaplastic thyroid cancer, elevated expression of PDL1 in tumor cells can suppress the anti-tumor activity of T cells, leading to cancer progression. PD1/PDL1 inhibitors, such as pembrolizumab and nivolumab, have shown efficacy in some ATC patients, especially those with positive PDL1 expression. Approximately 22%-29% of ATC tumor samples have been reported to express PD-L1 117. A positive response to combined therapy with BRAF inhibitors and PD-1 inhibitors has been observed in ATC patients with BRAF mutation 118. The underlying mechanism behind the observed immune activation in ATC is unclear. The response to PD-1 blockade is associated with higher levels of neoantigens created by chronic exposure to mutagens, such as tobacco smoke or UV light, or by defects in DNA repair, as found in microsatellite instability colorectal cancer 119. In a multicenter study of prospectively and retrospectively collected clinical data and tissue samples, PD-L1 expression in poorly differentiated thyroid cancers and ATC has been investigated. Variable expression was found in both aggressive thyroid malignancies, with higher expression in ATC than in other poorly differentiated cancers 120.

Spartalizumab, a PD-1 monoclonal antibody, showed good tolerability and promising clinical activity in ATC patients. Durable responses were observed in a subset of patients, including complete and partial responses, even in patients without BRAF mutations. PD-L1 expression and the presence of an inflamed tumor microenvironment were associated with better treatment responses. Although the underlying mechanism is still unclear, these findings suggest that PD-1 blockade may be a promising treatment option for ATC patients, especially those with positive PD-L1 expression 121.

A greater response to these treatments has been observed in patients with evidence of an inflamed tumor microenvironment at baseline, as suggested by higher PD-L1 expression, presence of CD8+ lymphocytes, and expression of genes involved in IFNγ signaling. An association between baseline PD-L1 expression and survival was also observed, with a median OS not reached in the subset of patients with PD-L1 expression ≥ 1% at baseline and poor survival in those who progressed to immunotherapy treatment 122.

#### 5.1.2 Beta-catenin, PAX8, and E-cadherin

Beta-catenin is a protein that plays a significant role in regulating cell growth and proliferation. Abnormal activation of the Wnt/β-catenin signaling pathway has been observed in anaplastic thyroid cancer. Beta-catenin overexpression is associated with uncontrolled cell proliferation, invasion, and metastasis. Beta-catenin expression has been observed to vary depending on the subtype of thyroid cancer, as demonstrated by immunohistochemical techniques 123.

PAX8 and E-cadherin are proteins that play important roles in thyroid tissue differentiation and development. PAX8 is a transcription factor essential for thyroid embryonic development and is expressed in thyroid follicular cells and carcinomas derived from these cells. On the other hand, E-cadherin is a cell adhesion molecule expressed in epithelial cells and regulates cell adhesion, migration, and polarity. Loss of E-cadherin expression has been associated with epithelial-mesenchymal transition, a significant pathological mechanism in thyroid carcinoma tumor progression 124.

Regarding beta-catenin expression, in well-differentiated thyroid cancers, reactivity is primarily localized in the lateral membrane, whereas in poorly differentiated carcinomas or squamous lineage, reactivity is continuously presented along the cell membrane, called "fishnet pattern," and has a weaker intensity, punctate intracytoplasmic positivity near the nuclei, and nuclear positivity. In one study, the rate of membrane positivity for β-catenin in ATC patients was 82.8% 125.

Regarding PAX8, loss of its expression is more typical of poorly differentiated thyroid cancers, and ATC has been observed to have loss or discontinuous patterns of E-cadherin expression 127.

In summary, the expression pattern of β-catenin and loss of expression of PAX8 and E-cadherin are useful for detecting poorly differentiated thyroid carcinoma subtypes such as ATC; therefore, their use in an immunohistochemical panel is recommended for a more comprehensive diagnosis of ATC.

#### 5.1.3. Vascular Endothelial Growth Factor (VEGF)

Angiogenesis is a fundamental process in tumor development and progression. VEGF is one of the main inducers of new blood vessel formation in the tumor microenvironment. In anaplastic thyroid cancer, VEGF overexpression correlates with worse prognosis and greater tumor aggressiveness.

Some studies have shown that VEGF levels are significantly higher in ATC samples compared to other forms of thyroid cancer and normal thyroid tissue. This VEGF overexpression may contribute to the formation of new blood vessels feeding tumor growth, facilitating cancer spread to other parts of the body 128. However, in one study, immunohistochemical staining of VEGF in thyroid tumor cells was significantly more frequent in well-differentiated thyroid cancers compared to poorly differentiated thyroid cancers, such as ATC. This suggests that this factor could play an important role in the early stages of carcinogenesis by supporting tumor growth through new blood vessel formation and exerting an immunosuppressive function 129.

Inhibition of VEGF or its receptor (VEGFR) has been explored as a potential therapeutic strategy in ATC treatment. Some preclinical studies have shown that VEGF inhibition can reduce tumor growth and angiogenesis in experimental models of ATC. Furthermore, clinical trials have been conducted to evaluate the efficacy of anti-VEGF drugs, such as bevacizumab, in ATC patients, although the results so far have been mixed, and more research is needed to determine their efficacy and safety in this specific context 130.

#### 5.1.4. Fibroblast Growth Factor Receptors (FGFRs)

Fibroblast growth factor receptors are a family of tyrosine kinase receptors that play a crucial role in regulating cell growth, differentiation, and survival. In ATC, mutations and overexpressions of FGFR have been observed to be associated with tumor progression and therapy resistance.

FGFR inhibition has been proposed as a potential therapeutic strategy in ATC treatment. Several preclinical studies have shown that the use of FGFR inhibitors can reduce tumor growth and invasion in experimental models of ATC 131.

Lenvatinib is a tyrosine kinase inhibitor that acts by blocking several signaling pathways involved in cell growth and proliferation. This includes the inhibition of VEGFR and FGFR. In the case of ATC, where treatment options are limited, and the disease is often resistant to conventional therapy, lenvatinib has shown some efficacy in clinical studies. It has been used both as monotherapy and in combination with other drugs, such as immune checkpoint inhibitors (e.g., PD-1/PD-L1 inhibitors), with varying results 132.

### 5.2. Serological Biomarkers

Thyroglobulin, calcitonin, and CEA (carcinoembryonic antigen) are tumor markers that can be useful in the management and monitoring of anaplastic thyroid carcinoma (ATC), although their specific utility may vary depending on the patient and other individual disease characteristics.

#### 5.2.1. Thyroglobulin

Thyroglobulin is a protein produced by the follicular cells of the thyroid gland. Under normal conditions, thyroglobulin is secreted into the blood in very small amounts. However, in the presence of thyroid cancer, thyroglobulin levels may increase. In anaplastic thyroid carcinoma, thyroglobulin levels are generally low or undetectable. This is because ATC is a poorly differentiated form of thyroid cancer that usually does not produce significant amounts of thyroglobulin. Due to the low expression of thyroglobulin in ATC, it has limited utility as a biomarker in the diagnosis and monitoring of the disease, unlike more differentiated thyroid cancers, where elevated serum thyroglobulin levels may indicate the presence of residual or recurrent disease 133.

#### 5.2.2. Calcitonin

Calcitonin is a hormone produced by the parafollicular cells of the thyroid gland (C cells). Calcitonin levels may increase in the presence of medullary thyroid carcinoma as it originates from C cells. In ATC, calcitonin levels are generally not elevated, as ATC is very poorly differentiated or usually derives from follicular cells. Therefore, measuring calcitonin levels is usually not useful in the diagnosis or monitoring of ATC, but may be relevant in the evaluation of other types of thyroid cancer, such as medullary carcinoma 134.

#### 5.2.3. CEA

CEA is a protein found in normal amounts in fetal tissues and in very small amounts in adult tissues. CEA levels may be elevated in various types of cancer, including thyroid cancers. In ATC, CEA levels may be elevated in some patients, but this is not uniform, and not all patients with ATC will have elevated CEA levels. Determining CEA levels may be useful in monitoring ATC and assessing response to treatment, but it is not specific or sensitive for this type of cancer and should be interpreted along with other clinical findings and diagnostic tests 135.

Therefore, ATC, being a poorly differentiated and highly aggressive cancer, limits the utility of certain serological biomarkers that are commonly used in differentiated thyroid cancers. For example, thyroglobulin and calcitonin, which are specific markers of functional thyroid cells, tend to be sparsely expressed or absent in ATC due to its poorly differentiated nature. Additionally, CEA, although often elevated in various types of cancer, lacks specificity, and its elevation may not be exclusive to ATC. While CEA may provide some diagnostic support, its utility is limited due to its lack of specificity.

### 5.3. Genetic and Molecular Biomarkers

Genetic mutations play a fundamental role in the development and progression of ATC, and their identification can have important clinical and therapeutic implications. Constitutive activation of the MAPK signaling pathway plays a crucial role in the carcinogenesis of various types of thyroid carcinoma. This pathway involves tyrosine kinase receptors such as VEGFR, RET, ALK, and TRK, as well as proteins like RAS, RAF, MEK, and ERK. In thyroid cancer, genetic alterations in the MAPK pathway are highly prevalent and mutually exclusive. These alterations lead to changes in cell proliferation, differentiation, and survival, contributing to the pathogenesis of various subtypes of thyroid carcinoma. The detection of these genetic alterations has become more precise and comprehensive thanks to next-generation sequencing (NGS) technology, allowing simultaneous analysis of multiple genes in a single test, providing a comprehensive picture of the genomic profile of a tumor quickly and efficiently.

#### 5.3.1. Mutations in the TERT Promoter

Mutations in the TERT promoter are significant findings in the context of thyroid carcinoma. TERT, or telomerase, is an enzyme responsible for maintaining the length of telomeres, the protective structures at the ends of chromosomes. Telomerase activation through mutations in the TERT promoter may contribute to cell immortalization and uncontrolled proliferation, key features of tumorigenesis.

In well-differentiated thyroid carcinoma, TERT promoter mutations are detected infrequently. In contrast, poorly differentiated carcinoma and anaplastic carcinoma show significantly higher rates of TERT promoter mutation, with a prevalence of 40% in poorly differentiated carcinoma and 73% in ATC 136 This high frequency of TERT promoter mutations suggests a fundamental role in the progression towards more aggressive and less differentiated forms of thyroid cancer.

It is important to note that TERT promoter mutations tend to be subclonal in differentiated carcinomas, meaning they are present in only a portion of tumor cells, while they are clonal in poorly differentiated thyroid carcinoma (PDTC) and ATC, indicating they are present in all or most tumor cells. This pattern suggests that TERT promoter mutations may be an early event in the tumorigenesis of PDTC and ATC, and may play a crucial role in their development and progression 137.

#### 5.3.2 Mutations in the BRAF and RAS Genes

Mutations in the BRAF and RAS genes remain critical in the development of poorly differentiated thyroid carcinoma and anaplastic thyroid carcinoma. In the case of PDTC, these mutations occur in approximately 33% and 45% of cases, respectively. On the other hand, in ATC, they are present in around 29% and 23% of cases, respectively. The BRAF V600E mutation is one of the most common genetic alterations in ATC and is present in approximately 40-50% of cases. This mutation activates the MAPK signaling pathway, promoting cell growth and survival 138.

The frequency of these mutations may vary depending on how the disease is defined and classified. For example, when using the criteria proposed by Turin to define poorly differentiated thyroid carcinoma, which include a solid growth pattern, absence of nuclear features of papillary thyroid carcinoma, and presence of necrosis with a mitotic index of ≥3/10 high-power fields (HPF) or convoluted nuclei, a high frequency of RAS mutations (42-64%) and a low frequency of BRAF V600E mutations (6-9%) are observed 139. However, when different criteria, such as those from the Memorial Sloan Kettering Cancer Center (MSKCC), based solely on necrosis or a mitotic index of ≥5/10 HPF, regardless of architectural pattern and nuclear features, are used, both tumors that meet the Turin criteria and an additional set of tumors that do not meet them are found. This latter subgroup of tumors tends to have a high frequency (67-78%) of BRAF mutations and a low rate (6-13%) of RAS mutations 140.

These differences in mutation frequency according to classification criteria underline the complexity and heterogeneity of ATC and suggest that different subpopulations may have distinctive genetic profiles, which could affect both prognosis and treatment options.

#### 5.3.3 Alterations in TP53

Inactivating mutations in TP53 are considered a hallmark genetic feature of anaplastic thyroid carcinoma. This tumor suppressor gene is essential in regulating the cell cycle and DNA repair, so its inactivation or mutation can lead to uncontrolled cell proliferation and tumor progression.

In the context of well-differentiated thyroid carcinoma, mutations in TP53 are quite rare. This low frequency reflects the less aggressive and more indolent nature of these tumors, while they are highly prevalent in ATC, detected in approximately 73% of cases 141. This high incidence underscores the crucial role of TP53 dysfunction in malignant transformation and aggressiveness of ATC.

In addition to TP53, ATC also shows mutations in other tumor suppressor genes, such as ATM, RB1, MEN1, NF1, and NF2. These additional mutations further contribute to cell cycle dysregulation and cell survival, thus promoting tumor progression and therapy resistance in these poorly differentiated thyroid cancer types 142.

#### 5.3.4. Alterations in PIK3CA

Alterations in the PIK3CA gene, which encodes the alpha subunit of phosphatidylinositol 3-kinase (PI3K), are observed in a significant proportion of ATC cases. Abnormal activation of the PI3K/AKT/mTOR signaling pathway due to mutations in PIK3CA can promote tumor growth, angiogenesis, and resistance to apoptosis. Mutations affecting components of the PIK3CA-AKT-mTOR pathway have been observed in 2-11% of poorly differentiated thyroid cancers and in 12-39% of ATC. These mutations involve not only well-known genes like PIK3CA and PTEN but also other members of the pathway, including PIK3C2G, PIK3CG, PIK3C3, PIK3R1, PIK3R2, AKT3, TSC1, TSC2, and MTOR 143.

#### 5.3.5 Mutations in SWI/SNF, HMT, and the MMR Pathway

The SWI/SNF chromatin remodeling complex is another important component of epigenetic genome control and plays a crucial role in regulating gene expression. Genes encoding components of the SWI/SNF complex, such as ARID1A or ARID1B, can alter chromatin structure and lead to changes in gene expression that favor tumor development. These are found in approximately 36% of anaplastic thyroid carcinomas and 6% of poorly differentiated thyroid carcinomas 144.

Histone methyltransferase (HMT) is an enzyme that adds methyl groups to histones, thereby modifying chromatin structure and regulating gene expression. Mutations in genes encoding histone methyltransferases can alter the epigenetic regulation of genes and promote carcinogenesis. These mutations are also more frequently found in ATC, at an approximate rate of 24% 145.

Additionally, alterations in the DNA mismatch repair (MMR) pathway, an essential DNA repair mechanism that corrects errors occurring during DNA replication, ensuring genomic integrity, have been found. These include genomic alterations such as MSH2, MSH6, and MLH1, in 12% of ATC and 2% of poorly differentiated cancers. Tumors with MMR mutations showed a hypermutator phenotype, with a higher number of mutations compared to tumors without these mutations. Dysfunction in the MMR pathway is associated with a microsatellite instability (MSI) phenotype, characterized by the presence of DNA sequence repeat errors known as microsatellites. Detection of MSI in ATC tumors may have therapeutic implications, as tumors with MSI have been shown to respond differently to certain treatments, such as immune checkpoint inhibitor immunotherapy 146.

#### 5.3.6 RET/PTC Rearrangements and Mutations in RET

The RET/PTC rearrangements and mutations in the RET gene are genetic events that are considered relatively rare in anaplastic thyroid carcinoma (ATC). However, they have been observed to be present in a small proportion of ATC cases, indicating their potential contribution to the pathogenesis of this disease.

These genetic alterations involve changes in the structure or function of the RET gene, which encodes a tyrosine kinase receptor essential for the regulation of cell growth and differentiation. Activation of the RET signaling pathway can lead to uncontrolled cell growth and prolonged survival of cancer cells, thereby contributing to the development and progression of anaplastic thyroid carcinoma. While these genetic alterations may be less common compared to mutations such as BRAF or RAS in ATC, their presence suggests the genetic diversity and molecular complexity of this disease 147.

#### 5.3.7. Amplification and Mutations in MET

Amplification and mutations in the MET gene are relatively common findings in anaplastic thyroid carcinoma (ATC). The MET receptor tyrosine kinase is a cell membrane protein that plays a crucial role in regulating cell growth, proliferation, and survival. When MET undergoes amplification or mutations resulting in abnormal activation, it can lead to a series of events that promote cancer invasion and metastasis. Additionally, aberrant activation of MET may confer resistance to targeted treatments, such as tyrosine kinase inhibitors 148.

#### 5.3.8. Mutations in the EGFR Gene

EGFR, also known as HER1 or ErbB1, is a cell membrane protein that belongs to the family of receptor tyrosine kinases. Its activation triggers a cascade of intracellular events that promote cell growth and survival, among other processes.

In the context of ATC, mutations in EGFR can abnormally activate the receptor signaling pathway, resulting in increased proliferation and resistance to apoptosis of cancer cells. This could significantly contribute to the aggressiveness and rapid progression of the tumor. However, it is important to note that the frequency of these mutations in ATC is low compared to other types of cancer where EGFR mutations are more prevalent, such as non-small cell lung cancer. In lung cancer, EGFR mutations have been associated with favorable clinical responses to EGFR inhibitors, such as erlotinib or gefitinib. Therefore, the identification of EGFR mutations in patients with ATC could suggest the possibility of using specific EGFR-targeted therapies as part of the treatment. However, further studies are needed to fully understand the role of EGFR mutations in ATC and their clinical relevance in terms of prognosis and treatment response 149.

Therefore, mutations in the BRAF and RAS genes remain consistent throughout the development of thyroid cancer, from well-differentiated forms to poorly differentiated and anaplastic carcinomas, serving as early drivers of the disease. On the other hand, other mutations such as those in the TERT promoter, TP53, PIK3CA, SWI/SNF complex, and tumor suppressor genes, among others, accumulate progressively during tumor dedifferentiation, contributing to the progression towards more aggressive forms of thyroid cancer such as ATC.

### 5.4. Circulating Tumor Cells and MicroRNAs

As it was the case with other thyroid cancer subtypes, liquid biopsy has emerged as a promising tool in the study and management of ATC, providing information about the disease in a less invasive manner than traditional tissue biopsies. The quantification of the number of circulating tumor cells present in the blood sample can offer information about tumor burden and disease extent, as the presence of CTCs is associated with a worse prognosis. Additionally, in CTCs, the expression of specific tumor cell markers, such as EGFR, MET tyrosine kinase receptor, and other markers associated with tumor aggressiveness, is analyzed. On the other hand, circulating DNA sequencing can reveal specific genetic mutations associated with ATC, such as mutations in the BRAF, RAS, and TP53 genes. The quantification and analysis of these mutations can aid in the diagnosis and risk stratification of patients with ATC 150.

Regarding miRNAs, specific miRNA expression profiles have been identified in ATC. MiRNA dysregulation has been associated with thyroid cancer progression, suggesting their involvement in tumor biology. Mutations in the miRNA biogenesis machinery are related to thyroid carcinoma progression, affecting the maturation and processing of miRNAs and their regulatory function. It has been proposed that alterations in miRNA expression due to these mutations underlie changes in proliferation, migration, and epithelial-mesenchymal transition in thyroid cancer, which may influence patient survival.

Misiak *et al.* 151 conducted a comprehensive analysis to identify associations of microRNA and messenger RNA targets relevant to anaplastic thyroid carcinoma. The results obtained suggest that ATC is characterized by significant miRNA expression dysregulation, indicating that the extreme reorganization of the mRNA transcriptome in ATC is partially driven by this miRNA dysregulation. This alteration affects various key oncogenes and tumor suppressors, suggesting that miRNA-dependent control in gene expression is a crucial factor in the severe dysregulation observed in ATC. Among the analyzed miRNAs, miR-146-5p and miR-210-3p were observed to be associated with promoting aggressive behavior in tumor cells. Conversely, miRNAs such as miR-21-5p were associated with dedifferentiation and transdifferentiation similar to epithelial-mesenchymal transition, a distinctive feature of ATC.

## 6. Future Directions

### 6.1. Molecular Biology Techniques

Spatial transcriptomics (SPT) and single-cell RNA sequencing (scRNA-seq) have revolutionized the analysis of cellular and molecular biology by providing unprecedented details on gene expression in spatial contexts and at the individual cell level. SPT enables the mapping of gene expression across tissue sections, offering a detailed view of how RNA profiles are distributed and vary within the tumor microenvironment. On the other hand, scRNA-seq provides an in-depth understanding of cellular heterogeneity by analyzing the RNA of individual cells, revealing the molecular diversity among seemingly similar cells. However, despite the significant advancements these technologies have brought, the expertise of pathologists remains crucial.

Althoug most patients with papillary thyroid carcinoma (PTC) have a favorable prognosis, a significant proportion develop a more aggressive form of the disease due to factors such as tumor dedifferentiation, the presence of atypical follicular cells (AFCs), and immune cells, leading to notable cellular heterogeneity. Traditional pathology techniques have limitations in assessing this cellular heterogeneity in PTC. Therefore, high-precision methods like single-cell RNA sequencing (scRNA-seq) and spatial transcriptomics (SPT) are being increasingly used. In a recent study 152 tissues from four PTC patients were collected and SPT was employed along with clinical pathological identification methods to analyze cellular heterogeneity. RNA-clinical signature genes for each cell type were identified, allowing precise mapping of specific cell distribution regions in tissue sections. AFCs were recognized as a transitional state towards tumor cells. Key ligand-receptor interactions were discovered, playing a crucial role in the transition from typical follicular cells to tumor cells, correlating with poorer prognosis. Additionally, the reduction of certain immune interactions contributed to tumorigenesis and increased cellular heterogeneity.

This approach, integrating RNA-based clustering with clinical pathology information, enhances the accuracy of spatial transcriptome analysis and provides valuable insights for prognosis evaluation in PTC patients.

ATC and PTC both originate from follicular epithelial cells, but ATC is associated with a much poorer prognosis and often shows resistance to conventional treatments. A study 153 has shown that immunotherapy is more effective in ATC compared to advanced PTC. To investigate this, researchers used scRNA-Seq to create a detailed single-cell atlas of thyroid cancer and compared the tumor microenvironment components between ATC and PTC, including malignant, stromal, and immune cells. They discovered that CXCL13+ T lymphocytes were more prevalent in ATC, potentially enhancing the tumor's sensitivity to immunotherapy.

### 6.2 New Targeted Therapies

There are ongoing clinical trials evaluating the efficacy and safety of new targeted therapies. Regarding tyrosine kinase inhibitors, a phase 1-2 clinical trial 154 evaluated selpercatinib in patients with medullary thyroid cancer with a RET mutation and patients with RET fusions thyroid cancer. The results showed that selpercatinib had a high objective response rate (79%) and good progression-free survival (64% at one year), with mostly low-grade side effects. A recent Phase 3 clinical trial has been published concluding that treatment with selpercatinib resulted in superior progression-free survival and treatment-free survival compared to other therapies such as cabozantinib or vandetanib in patients with RET-mutant medullary thyroid cancer 155.

Another RET inhibitor, pralsetinib, which has shown efficacy in lung cancer, has also been studied in patients with thyroid cancer with this genetic alteration. One phase 1-2 study 156 demonstrated the efficacy and safety of pralsetinib in patients with medullary thyroid cancer, with response rates of 71% in treatment-naive patients, 60% in those previously treated with cabozantinib and/or vandetanib, and 89% in patients with RET fusions. Inhibitors such as larotrectinib and entrectinib have shown promising results in clinical trials, particularly in patients with NTRK fusions and metastatic thyroid cancer 157,158.

As for immunotherapy, PD-1/PD-L1 inhibitors, such as pembrolizumab and nivolumab, are being investigated in combination with other targeted therapies, like BRAF inhibitors, to improve the immune response in patients with refractory thyroid cancer. These tumors, often resistant to conventional treatments, may benefit from the combination of therapies to enhance antitumor activity, leveraging both the ability of targeted therapies to inhibit tumor growth and the activation of the immune system to eliminate cancer cells 159.

## 7. Conclusions

In conclusion, the review emphasizes the indispensable role of biomarkers in refining the management of thyroid cancer towards individualized therapy. It demonstrates that biomarkers are key for detecting aggressive anaplastic thyroid cancer, prognosticating outcomes for papillary thyroid cancer, and selecting precise treatments for medullary thyroid cancer, especially for cases with RET gene mutations. This targeted approach significantly improves diagnostic accuracy, optimizes treatment effectiveness, and enhances overall patient prognosis. Furthermore, the review highlights the necessity for detailed genetic profiling in follicular thyroid cancer to devise customized treatment plans. Ultimately, it advocates for expanded research into biomarkers and personalized therapy options to advance patient care in thyroid cancer.

## Figures and Tables

**Table 1:** This table provides an overview of critical biomarkers for Papillary Thyroid Cancer (PTC), categorized by type: histological, serological, genetic, and circulating. These markers are essential for diagnosing, prognosticating, and determining the treatment course for PTC.

Marker Type	Marker Name	Marker Utility	References
Histological marker	HBME1	Differentiation of papillary thyroid carcinoma from benign lesions; expressed in papillary carcinoma and its variants.	12, 13
	CK19	High sensitivity for PTC; useful in differentiating PTC from benign lesions.	14, 15, 16
Serological marker	Thyroglobulin (TG)	Marker for residual or recurrent disease following initial treatment in PTC.	17, 18
Genetic marker	BRAF V600E Mutations	Associated with high-risk clinical features and worse prognosis in PTC. Options for targeted therapy in PTC and anaplastic cancers.	26, 27, 28, 29, 30
	RET/PTC Rearrangements	Specific for PTC, common in radiation-associated tumors. Important for diagnosis and targeted therapies.	21, 22, 23, 24, 25
	RAS Mutations	Common in follicular variants of PTC, involved in tumor dedifferentiation and aggressiveness.	32, 33, 34
	TERT Mutations	Indicator of persistent disease and prognosis in PTC. Associated with aggressive behavior and distant metastases.	35, 36, 37, 38
	NTRK Fusions	Involved in the development and progression of PTC. Potential therapeutic targets for directed therapies.	39, 40, 41, 42
Circulating biomarkers	CTCs (Circulating Tumor Cells)	Indicative of tumor progression and metastasis; correlated with aggressive tumor features and treatment response in PTC.	44, 45, 46, 47, 48
	ctDNA (Circulating Tumor DNA)	Potential for non-invasive diagnosis, monitoring of progression, and treatment response in PTC.	49, 50, 51, 52, 53, 54, 55
	MicroRNAs	Markers for diagnosis, prognosis, and recurrence prediction in PTC. Distinguish between thyroid cancer types and predict tumor invasion.	56, 57, 58

**Table 2:** This table outlines the diverse array of biomarkers relevant to the diagnosis, prognosis, and treatment of Thyroid Follicular Carcinoma (FTC).

Marker Type	Marker Name	Marker Utility	References
Histological marker	HBME-1, Galectin-3, CK19, CD56	Differentiation of FTC from benign follicular-patterned thyroid lesions and other thyroid cancers.	61, 62, 63, 64, 65, 66, 67
Serological marker	Thyroglobulin (TG)	Monitoring and detecting residual or recurrent disease after treatment.	68
Genetic marker	RAS mutations	Found in approximately 40% of FTCs, indicating potential aggressiveness and higher mortality.	69, 70
	PAX8-PPAR gamma 1 rearrangement	Distinguishes between follicular adenoma and carcinoma, associated with a more favorable prognosis.	71, 72, 73
	TERT mutations	Associated with aggressive tumor behaviors and poor clinical outcomes, including recurrence and mortality.	74, 75
	PTEN mutations	Related to familial neoplastic syndrome and a higher risk of recurrence and metastasis in FTC.	76
	TSHR mutations	Found in hyperfunctioning nodules and cancers, associated with a higher risk of recurrence and metastasis.	77, 78, 79
	EIF1AX mutations	Associated with benign and malignant follicular thyroid neoplasms, linked to a more favorable prognosis.	80
MicroRNA	miR-21, miR-181a-5p, miR-197, miR-346	Differentiation between FTC and PTC, and potential indicators of FTC aggressiveness and prognosis.	83, 84, 85, 86, 8

**Table 3:** This table provides a detailed overview of the critical biomarkers used in the diagnosis, prognosis, and therapeutic management of Medullary Thyroid Carcinoma (MTC).

Marker Type	Marker Name	Marker Utility	References
Histological Markers	Calcitonin	Essential for MTC diagnosis; high immunoreactivity indicates MTC origin. Presence in tumor suggests C-cell derivation.	99, 104
	CEA (Carcinoembryonic Antigen)	Used alongside calcitonin for monitoring MTC progression and recurrence. Less sensitive than calcitonin but provides prognostic value.	104, 105
	bcl-2, Ki-67, p53, C-erb-B2	Indicate tumor proliferation rate (Ki-67), apoptosis inhibition (bcl-2), tumor suppressor gene status (p53), and oncogene activation (C-erb-B2).	104
Serological Markers	Calcitonin	Gold standard for MTC diagnosis and monitoring. Elevated levels indicate MTC presence and progression.	105, 106, 107
	CEA	Supports diagnosis and monitoring, especially in conjunction with calcitonin. Elevated levels correlate with tumor burden and prognosis.	105, 107
Genetic Markers	RET Oncogene Mutations	Critical for identifying hereditary MTC within MEN2 syndrome. Predictive of disease aggressiveness and therapeutic response.	98, 110, 111
Circulating Biomarkers	CTCs (Circulating Tumor Cells)	Indicative of metastatic disease and survival rates. Offer insights into disease aggressiveness and potential for targeted therapy.	112, 113, 114, 115
Emerging Markers	MicroRNAs (miRNAs)	Under investigation for their roles in MTC pathogenesis, prognosis, and therapeutic targeting. Require further validation for clinical use.	(Referenced in general discussion)

**Table 4:** This detailed table encapsulates the vital biomarkers involved in the management of Anaplastic Thyroid Cancer (ATC), highlighting their diagnostic and prognostic significance as well as their role as potential therapeutic targets.

Marker Type	Marker Name	Marker Utility	References
Histological Markers	PDL-1	Associated with immune evasion; predictive of response to PD-1/PDL-1 inhibitors.	117, 118, 119, 120, 121, 122
	Beta-catenin, PAX8, E-cadherin	Indicators of cell proliferation, differentiation, and adhesion; involved in tumor progression.	123, 124, 125, 127
	VEGF	Promotes angiogenesis; correlated with tumor aggressiveness and metastasis.	128, 129, 130
	FGFRs	Involved in cell growth and survival; target for FGFR inhibitors.	131, 132
Serological Markers	Thyroglobulin	Not typically elevated in ATC due to its poorly differentiated nature.	133
	Calcitonin	Not useful in ATC diagnosis or monitoring.	134
	CEA	May be elevated in some ATC cases; used for monitoring treatment response.	135
Genetic and Molecular Markers	TERT Promoter Mutations	Associated with aggressive tumor behavior; prevalent in ATC.	136, 137
	BRAF, RAS Mutations	Common in ATC; early drivers of thyroid carcinogenesis.	138, 139, 140
	TP53, PIK3CA Mutations	Involved in tumor progression and resistance to therapy.	141, 142, 143
	SWI/SNF, HMT, MMR Pathway Alterations	Affect gene expression and DNA repair mechanisms; associated with advanced thyroid cancer.	144, 145, 146
	RET/PTC Rearrangements, MET and EGFR Mutations	Rare but contribute to pathogenesis; potential targets for therapy.	147, 148, 149
Circulating Biomarkers	CTCs, miRNAs	Provide information on tumor burden and disease extent; associated with prognosis and potential therapeutic targets.	150, 151

## References

[1] Denaro N, Lo Nigro C, Russi EG, Merlano M (2013). The role of chemotherapy and latest emerging target therapies in anaplastic thyroid cancer. OncoTargets Ther.

[2] Maniakas A, Zafereo M, Cabanillas ME (2022). Anaplastic thyroid cancer. Endocrinol Metab Clin North Am.

[3] Bergdorf K, Ferguson DC, Mehrad M, Ely K, Stricker T, Weiss VL (2019). Papillary thyroid carcinoma behavior: clues in the tumor microenvironment. Endocr Relat Cancer.

[4] Lam AK (2022). Histopathological assessment for papillary thyroid carcinoma. In: Methods Mol Biol. New York: Springer US.

[5] Jaber T, Dadu R, Hu MI (2021). Medullary thyroid carcinoma. Curr Opin Endocrinol Diabetes Obes.

[6] Matrone A, Gambale C, Prete A, Elisei R (2022). Sporadic medullary thyroid carcinoma: towards a precision medicine. Front Endocrinol.

[7] Aschebrook-Kilfoy B, Grogan RH, Ward MH, Kaplan E, Devesa SS (2013). Follicular thyroid cancer incidence patterns in the United States, 1980-2009. Thyroid.

[8] Milano AF (2018). Thyroid cancer: 20-year comparative mortality and survival analysis of six thyroid cancer histologic subtypes by age, sex, race, stage, cohort entry time-period and disease duration (SEER*Stat 8.3.2) a systematic review of 145,457 cases for diagnosis years 1993-2013. J Insur Med.

[9] Xu J, Zhang Y, Liu J, Qiu S, Wang M (2021). A population-based study of the three major variants of papillary thyroid carcinoma. J Int Med Res.

[10] Cartwright S, Fingeret A (2020). Contemporary evaluation and management of tall cell variant of papillary thyroid carcinoma. Curr Opin Endocrinol Diabetes Obes.

[11] Prasad ML, Pellegata NS, Huang Y, Nagaraja HN, de la Chapelle A, Kloos RT (2005). Galectin-3, fibronectin-1, CITED-1, HBME1 and cytokeratin-19 immunohistochemistry is useful for the differential diagnosis of thyroid tumors. Mod Pathol.

[12] Cheung CC, Ezzat S, Freeman JL, Rosen IB, Asa SL (2001). Immunohistochemical diagnosis of papillary thyroid carcinoma. Mod Pathol.

[13] Nasr MR, Mukhopadhyay S, Zhang S, Katzenstein A-LA (2006). Immunohistochemical markers in diagnosis of papillary thyroid carcinoma: utility of HBME1 combined with CK19 immunostaining. Mod Pathol.

[14] Viana A de OR, Gonçalves Filho J, Francisco ALN, Pinto CAL, Kowalski LP (2020). Ki-67 and CK-19 are predictors of locoregional recurrence in papillary thyroid carcinoma. Acta Otorhinolaryngol Ital.

[15] Xia S, Wang C, Postma EL, Yang Y, Ni X, Zhan W (2017). Fibronectin 1 promotes migration and invasion of papillary thyroid cancer and predicts papillary thyroid cancer lymph node metastasis. OncoTargets Ther.

[16] Arcolia V, Journe F, Renaud F (2017). Combination of galectin-3, CK19 and HBME-1 immunostaining improves the diagnosis of thyroid cancer. Oncol Lett.

[17] Spencer CA, LoPresti JS (2008). Technology insight: measuring thyroglobulin and thyroglobulin autoantibody in patients with differentiated thyroid cancer. Nat Clin Pract Endocrinol Metab.

[18] Lin J-D (2008). Thyroglobulin and human thyroid cancer. Clin Chim Acta.

[19] Abdullah MI, Junit SM, Ng KL, Jayapalan JJ, Karikalan B, Hashim OH (2019). Papillary thyroid cancer: genetic alterations and molecular biomarker investigations. Int J Med Sci.

[20] Cabanillas ME, McFadden DG, Durante C (2016). Thyroid cancer. Lancet.

[21] Capdevila J, Awada A, Führer-Sakel D, Leboulleux S, Pauwels P (2022). Molecular diagnosis and targeted treatment of advanced follicular cell-derived thyroid cancer in the precision medicine era. Cancer Treat Rev.

[22] Eszlinger M, Stewardson P, McIntyre JB (2022). Systematic population-based identification of NTRK and RET fusion-positive thyroid cancers. Eur Thyroid J.

[23] Hsiao SJ, Zehir A, Sireci AN, Aisner DL (2019). Detection of tumor NTRK gene fusions to identify patients who may benefit from tyrosine kinase (TRK) inhibitor therapy. J Mol Diagn.

[24] Zhao X, Kotch C, Fox E (2021). NTRK fusions identified in pediatric tumors: the frequency, fusion partners, and clinical outcome. JCO Precis Oncol.

[25] Haberecker M, Töpfer A, Melega F, Moch H, Pauli C (2023). A systematic comparison of pan-Trk immunohistochemistry assays among multiple cancer types. Histopathology.

[26] Xing M (2005). BRAF mutation in thyroid cancer. Endocr Relat Cancer.

[27] Johnson WT, Patel P, Hernandez A (2015). Langerhans cell histiocytosis and Erdheim-Chester disease, both with cutaneous presentations, and papillary thyroid carcinoma all harboring the BRAFV600E mutation. J Cutan Pathol.

[28] Al Hamad MA, Albisher HM, Al Saeed WR, Almumtin AT, Allabbad FM, Shawarby MA (2019). BRAF gene mutations in synchronous papillary thyroid carcinoma and Langerhans cell histiocytosis co-existing in the thyroid gland: a case report and literature review. BMC Cancer.

[29] Hanly EK, Rajoria S, Darzynkiewicz Z (2014). Disruption of mutated BRAF signaling modulates thyroid cancer phenotype. BMC Res Notes.

[30] Xu X, Prinz RA (2016). Targeting B-Raf kinase for thyroid cancer treatment: promise and challenge. Transl Cancer Res.

[31] Smith RA, Salajegheh A, Weinstein S, Nassiri M, Lam AK (2011). Correlation between BRAF mutation and the clinicopathological parameters in papillary thyroid carcinoma with particular reference to follicular variant. Hum Pathol.

[32] Rivera M, Ricarte-Filho J, Knauf J (2010). Molecular genotyping of papillary thyroid carcinoma follicular variant according to its histological subtypes (encapsulated vs infiltrative) reveals distinct BRAF and RAS mutation patterns. Mod Pathol.

[33] Xing M (2016). Clinical utility of RAS mutations in thyroid cancer: a blurred picture now emerging clearer. BMC Med.

[34] Gilani SM, Abi-Raad R, Garritano J, Cai G, Prasad ML, Adeniran AJ (2021). RAS mutation and associated risk of malignancy in the thyroid gland: an FNA study with cytology-histology correlation. Cancer Cytopathol.

[35] Chen B, Shi Y, Xu Y, Zhang J (2020). The predictive value of coexisting BRAFV600E and TERT promoter mutations on poor outcomes and high tumour aggressiveness in papillary thyroid carcinoma: a systematic review and meta-analysis. Clin Endocrinol (Oxf).

[36] Cao J, Zhu X, Sun Y, Li X, Yun C, Zhang W (2022). The genetic duet of BRAF V600E and TERT promoter mutations predicts the poor curative effect of radioiodine therapy in papillary thyroid cancer. Eur J Nucl Med Mol Imaging.

[37] Song YS, Lim JA, Choi H (2016). Prognostic effects of TERT promoter mutations are enhanced by coexistence with BRAF or RAS mutations and strengthen the risk prediction by the ATA or TNM staging system in differentiated thyroid cancer patients. Cancer.

[38] Gandolfi G, Ragazzi M, Frasoldati A, Piana S, Ciarrocchi A, Sancisi V (2015). TERT promoter mutations are associated with distant metastases in papillary thyroid carcinoma. Eur J Endocrinol.

[39] Pekova B, Sykorova V, Mastnikova K (2021). NTRK fusion genes in thyroid carcinomas: clinicopathological characteristics and their impacts on prognosis. Cancers (Basel).

[40] Haddad R, Elisei R, Hoff AO (2023). Diagnosis and management of tropomyosin receptor kinase fusion-positive thyroid carcinomas. JAMA Oncol.

[41] Lee Y, Hsu C, Lai C, Hang J (2021). NTRK-rearranged papillary thyroid carcinoma demonstrates frequent subtle nuclear features and indeterminate cytologic diagnoses. Cancer Cytopathol.

[42] Chu YH, Dias-Santagata D, Farahani AA (2020). Clinicopathologic and molecular characterization of NTRK-rearranged thyroid carcinoma (NRTC). Mod Pathol.

[43] Connal S, Cameron JM, Sala A (2023). Liquid biopsies: the future of cancer early detection. J Transl Med.

[44] Ringel MD, Ladenson PW, Levine MA (1998). Molecular diagnosis of residual and recurrent thyroid cancer by amplification of thyroglobulin messenger ribonucleic acid in peripheral blood. J Clin Endocrinol Metab.

[45] Pogliaghi G (2021). Liquid biopsy in thyroid cancer: from circulating biomarkers to a new perspective of tumor monitoring and therapy. Minerva Endocrinol.

[46] Xu JY, Handy B, Michaelis CL (2016). Detection and prognostic significance of circulating tumor cells in patients with metastatic thyroid cancer. J Clin Endocrinol Metab.

[47] Qiu Z-L, Wei W-J, Sun Z-K (2018). Circulating tumor cells correlate with clinicopathological features and outcomes in differentiated thyroid cancer. Cell Physiol Biochem.

[48] Winkens T, Pachmann K, Freesmeyer M (2014). The influence of radioiodine therapy on the number of circulating epithelial cells (CEC) in patients with differentiated thyroid carcinoma - a pilot study. Exp Clin Endocrinol Diabetes.

[49] Corcoran RB, Chabner BA (2018). Application of cell-free DNA analysis to cancer treatment. N Engl J Med.

[50] Chuang TCY, Chuang AYC, Poeta L, Koch WM, Califano JA, Tufano RP (2009). Detectable BRAF mutation in serum DNA samples from patients with papillary thyroid carcinomas. Head Neck.

[51] Lan X, Bao H, Ge X (2020). Genomic landscape of metastatic papillary thyroid carcinoma and novel biomarkers for predicting distant metastasis. Cancer Sci.

[52] Allin DM, Shaikh R, Carter P (2018). Circulating tumour DNA is a potential biomarker for disease progression and response to targeted therapy in advanced thyroid cancer. Eur J Cancer.

[53] Zane M, Agostini M, Enzo MV (2013). Circulating cell-free DNA, SLC5A8 and SLC26A4 hypermethylation, BRAFV600E: a non-invasive tool panel for early detection of thyroid cancer. Biomed Pharmacother.

[54] Brait M, Loyo M, Rosenbaum E (2012). Correlation between BRAF mutation and promoter methylation of TIMP3, RARβ2 and RASSF1A in thyroid cancer. Epigenetics.

[55] Lubitz CC, Zhan T, Gunda V (2018). Circulating BRAFV600E levels correlate with treatment in patients with thyroid carcinoma. Thyroid.

[56] Zhang H, Duan H-L, Wang S, Liu Y, Ding G-N, Lin R-X (2022). Epigenetic signature associated with thyroid cancer progression and metastasis. Semin Cancer Biol.

[57] Wang W, Zheng Z, Lei J (2023). CTC, ctDNA, and exosome in thyroid cancers: a review. Int J Mol Sci.

[58] Jiang K, Li G, Chen W (2020). Plasma exosomal miR-146b-5p and miR-222-3p are potential biomarkers for lymph node metastasis in papillary thyroid carcinomas. OncoTargets Ther.

[59] Rossi ED, Pantanowitz L, Hornick JL (2021). A worldwide journey of thyroid cancer incidence centred on tumour histology. Lancet Diabetes Endocrinol.

[60] Parameswaran R, Shulin Hu J, Min En N, Tan W, Yuan N (2017). Patterns of metastasis in follicular thyroid carcinoma and the difference between early and delayed presentation. Ann R Coll Surg Engl.

[61] Hofman V, Lassalle S, Bonnetaud C (2009). Thyroid tumours of uncertain malignant potential: frequency and diagnostic reproducibility. Virchows Arch.

[62] Donna A, Betta PG, Chiodera P (1997). Newly marketed tissue markers for malignant mesothelioma: immunoreactivity of rabbit AMAD-2 antiserum compared with monoclonal antibody HBME-1 and a review of the literature on so-called antimesothelioma antibodies. Hum Pathol.

[63] Chiu CG, Strugnell SS, Griffith OL (2010). Diagnostic utility of galectin-3 in thyroid cancer. Am J Pathol.

[64] Dunđerović D, Lipkovski JM, Boričic I (2015). Defining the value of CD56, CK19, Galectin 3 and HBME-1 in diagnosis of follicular cell derived lesions of thyroid with systematic review of literature. Diagn Pathol.

[65] Tastekin E, Keskin E, Can N (2019). CD56, CD57, HBME1, CK19, Galectin-3 and p63 immunohistochemical stains in differentiating diagnosis of thyroid benign/malign lesions and NIFTP. Pol J Pathol.

[66] Zargari N, Mokhtari M (2018). Evaluation of diagnostic utility of immunohistochemistry markers of TROP-2 and HBME-1 in the diagnosis of thyroid carcinoma. Eur Thyroid J.

[67] Nechifor-Boilă A, Cătană R, Loghin A, Radu TG, Borda A (2014). Diagnostic value of HBME-1, CD56, Galectin-3 and cytokeratin-19 in papillary thyroid carcinomas and thyroid tumors of uncertain malignant potential. Rom J Morphol Embryol.

[68] Spencer CA, Bergoglio LM, Kazarosyan M, Fatemi S, LoPresti JS (2005). Clinical impact of thyroglobulin (Tg) and Tg autoantibody method differences on the management of patients with differentiated thyroid carcinomas. J Clin Endocrinol Metab.

[69] Zhu Z, Gandhi M, Nikiforova MN, Fischer AH, Nikiforov YE (2003). Molecular profile and clinical-pathologic features of the follicular variant of papillary thyroid carcinoma. Am J Clin Pathol.

[70] Garcia-Rostan G, Zhao H, Camp RL (2003). ras mutations are associated with aggressive tumor phenotypes and poor prognosis in thyroid cancer. J Clin Oncol.

[71] Marques AR, Espadinha C, Catarino AL (2002). Expression of PAX8-PPARγ1 rearrangements in both follicular thyroid carcinomas and adenomas. J Clin Endocrinol Metab.

[72] Martelli ML, Iuliano R, Le Pera I (2002). Inhibitory effects of peroxisome proliferator-activated receptor γ on thyroid carcinoma cell growth. J Clin Endocrinol Metab.

[73] Armstrong MJ, Yang H, Yip L (2014). PAX8/PPARγ rearrangement in thyroid nodules predicts follicular-pattern carcinomas, in particular the encapsulated follicular variant of papillary carcinoma. Thyroid.

[74] Liu R, Xing M (2016). TERT promoter mutations in thyroid cancer. Endocr Relat Cancer.

[75] Haroon Al Rasheed MR, Xu B (2019). Molecular alterations in thyroid carcinoma. Surg Pathol Clin.

[76] Ngeow J, Mester J, Rybicki LA, Ni Y, Milas M, Eng C (2011). Incidence and clinical characteristics of thyroid cancer in prospective series of individuals with Cowden and Cowden-like syndrome characterized by germline PTEN, SDH, or KLLN alterations. J Clin Endocrinol Metab.

[77] Mon SY, Riedlinger G, Abbott CE (2018). Cancer risk and clinicopathological characteristics of thyroid nodules harboring thyroid-stimulating hormone receptor gene mutations. Diagn Cytopathol.

[78] Hoffmann S, Maschuw K, Hassan I (2006). Functional thyrotropin receptor attenuates malignant phenotype of follicular thyroid cancer cells. Endocrine.

[79] Agarwal S, Bychkov A, Jung C-K (2021). Emerging biomarkers in thyroid practice and research. Cancers (Basel).

[80] Gargano SM, Badjatia N, Nikolaus Y, Peiper SC, Wang Z-X (2021). Characterization and clinical significance of EIF1AX mutations and co-mutations. Acta Med Acad.

[81] Santiago K, Chen Wongworawat Y, Khan S (2020). Differential microRNA-signatures in thyroid cancer subtypes. J Oncol.

[82] Nana-Sinkam SP, Croce CM (2014). MicroRNA regulation of tumorigenesis, cancer progression and interpatient heterogeneity: towards clinical use. Genome Biol.

[83] Samsonov R, Burdakov V, Shtam T (2016). Plasma exosomal miR-21 and miR-181a differentiates follicular from papillary thyroid cancer. Tumour Biol.

[84] Stokowy T, Wojtaś B, Fujarewicz K, Jarząb B, Eszlinger M, Paschke R (2014). miRNAs with the potential to distinguish follicular thyroid carcinomas from benign follicular thyroid tumors: results of a meta-analysis. Horm Metab Res.

[85] Wojtas B, Ferraz C, Stokowy T (2014). Differential miRNA expression defines migration and reduced apoptosis in follicular thyroid carcinomas. Mol Cell Endocrinol.

[86] Ardizzone A, Calabrese G, Campolo M (2021). Role of miRNA-19a in cancer diagnosis and poor prognosis. Int J Mol Sci.

[87] Doghish AS, El-Mahdy HA, Ismail A (2023). Significance of miRNAs on the thyroid cancer progression and resistance to treatment with special attention to the role of cross-talk between signaling pathways. Pathol Res Pract.

[88] Nikiforov YE, Nikiforova MN (2011). Molecular genetics and diagnosis of thyroid cancer. Nat Rev Endocrinol.

[89] Scheffel RS, Dora JM, Maia AL (2021). BRAF mutations in thyroid cancer. Curr Opin Oncol.

[90] Wijewardene AA, Chehade M, Gild ML, Clifton-Bligh RJ, Bullock M (2021). Translational utility of liquid biopsies in thyroid cancer management. Cancers (Basel).

[91] Weng X, Yang Y, Cai Y (2022). Clinical significance of circulating tumor cells (CTCs) and survivin on predicting prognosis in thyroid cancer patients. Dis Markers.

[92] Cabanillas ME, Ryder M, Jimenez C (2019). Targeted therapy for advanced thyroid cancer: kinase inhibitors and beyond. Endocr Rev.

[93] Schubert L, Mariko ML, Clerc J, Huillard O, Groussin L (2023). MAPK pathway inhibitors in thyroid cancer: preclinical and clinical data. Cancers (Basel).

[94] Waguespack SG, Drilon A, Lin JJ (2022). Efficacy and safety of larotrectinib in patients with TRK fusion-positive thyroid carcinoma. Eur J Endocrinol.

[95] Cho JS, Kim HK (2022). Thyroglobulin levels as a predictor of papillary cancer recurrence after thyroid lobectomy. Anticancer Res.

[96] Ceolin L, Duval MA da S, Benini AF, Ferreira CV, Maia AL (2019). Medullary thyroid carcinoma beyond surgery: advances, challenges, and perspectives. Endocr Relat Cancer.

[97] Galofré JC, Santamaría Sandi J, Capdevila J, Navarro González E, Zafón Llopis C, Ramón Y Cajal Asensio T (2015). Consensus on the management of advanced medullary thyroid carcinoma on behalf of the Working Group of Thyroid Cancer of the Spanish Society of Endocrinology (SEEN) and the Spanish Task Force Group for Orphan and Infrequent Tumors (GETHI). Endocrinol Nutr.

[98] Wells SA, Asa SL, Dralle H, Elisei R, Evans DB, Gagel RF (2015). Revised American Thyroid Association guidelines for the management of medullary thyroid carcinoma. Thyroid.

[99] Zambudio AR, Rodríguez J, Riquelme J, Soria T, Canteras M, Parrilla P (2004). Prospective study of postoperative complications after total thyroidectomy for multinodular goiters by surgeons with experience in endocrine surgery. Ann Surg.

[100] Ríos A, Rodríguez JM, Galindo PJ, Montoya M, Tebar FJ, Sola J (2004). Utility of fine-needle aspiration for diagnosis carcinoma associated with multinodular goitre. Clin Endocrinol (Oxf).

[101] Dionigi G, Tanda ML, Piantanida E (2008). Medullary thyroid carcinoma: surgical treatment advances. Curr Opin Otolaryngol Head Neck Surg.

[102] Kaserer K, Scheuba C, Neuhold N, Weinhäusel A, Haas OA, Vierhapper H (2001). Sporadic versus familial medullary thyroid microcarcinoma: a histopathologic study of 50 consecutive patients. Am J Surg Pathol.

[103] Schröder S, Böcker W, Baisch H, Bürk CG, Arps H, Meiners I (1988). Prognostic factor in medullary thyroid carcinoma. Survival in relation to age, sex, stage, histology, immunocytochemistry and DNA content. Cancer.

[104] Bergholm U, Adami HO, Auer G, Bergstrom R, Backdahl M, Grimelius L (1989). Histopathological characteristics and DNA contents as prognostic factor in medullary thyroid carcinoma: a nationwide study in Sweden. Cancer.

[105] Hadoux J, Pacini F, Tuttle RM, Schlumberger M (2016). Management of advanced medullary thyroid cancer. Lancet Diabetes Endocrinol.

[106] Verbeek HH, de Groot JWB, Sluiter WJ, Muller Kobold AC, van den Heuvel ER, Plukker JT (2020). Calcitonin testing for detection of medullary thyroid cancer in people with thyroid nodules. Cochrane Database Syst Rev.

[107] Giannetta E, Guarnotta V, Altieri B, Sciammarella C, Guadagno E, Malandrino P (2020). Endocrine tumours: calcitonin in thyroid and extra-thyroid neuroendocrine neoplasms: the two-faced Janus. Eur J Endocrinol.

[108] Niederle MB, Scheuba C, Riss P, Selberherr A, Koperek O, Niederle B (2020). Early diagnosis of medullary thyroid cancer: are calcitonin stimulation tests still indicated in the era of highly sensitive calcitonin immunoassays?. Thyroid.

[109] Colombo C, Verga U, Mian C, Ferrero S, Perrino M, Vicentini L (2012). Comparison of calcium and pentagastrin tests for the diagnosis and follow-up of medullary thyroid cancer. J Clin Endocrinol Metab.

[110] Macerola E, Poma AM, Vignali P (2022). Predictive Biomarkers in Thyroid Cancer. Front Oncol.

[111] Hejax H A, Abuzaina I, Aldeen R N, Saad S (2022). Currently Used and New Molecular Markers for Thyroid Cancer Diagnosis. Middle East Journal of Cancer.

[112] Ferreira MM, Ramani VC, Jeffrey SS (2016). Circulating tumor cell technologies. Mol Oncol.

[113] Castro-Giner F, Aceto N (2020). Tracking cancer progression: from circulating tumor cells to metastasis. Genome Med.

[114] Sriramareddy SN, Hamoir E, Chavez M, Louis R, Beckers A, Willems L (2018). Tumor cells may circulate in medullary thyroid cancer patients independently of serum calcitonin. Endocr Relat Cancer.

[115] Ehlers M, Allelein S, Schwarz F (2018). Increased numbers of circulating tumor cells in thyroid cancer patients. Horm Metab Res.

[116] Haddad RI, Lydiatt WM, Ball DW (2015). Anaplastic thyroid carcinoma, version 2.2015. J Natl Compr Canc Netw.

[117] Wu H, Sun Y, Ye H, Yang S, Lee SL, de las Morenas A (2015). Anaplastic thyroid cancer: outcome and the mutation/expression profiles of potential targets. Pathol Oncol Res.

[118] De Leo S, Trevisan M, Fugazzola L (2020). Recent advances in the management of anaplastic thyroid cancer. Thyroid Res.

[119] Ng TSC, Gunda V, Li R (2021). Detecting immune response to therapies targeting PDL1 and BRAF by using ferumoxytol MRI and macrin in anaplastic thyroid cancer. Radiology.

[120] Adam P, Kircher S, Sbiera I (2021). FGF-receptors and PD-L1 in anaplastic and poorly differentiated thyroid cancer: evaluation of the preclinical rationale. Front Endocrinol (Lausanne).

[121] Capdevila J, Wirth LJ, Ernst T (2020). PD-1 blockade in anaplastic thyroid carcinoma. J Clin Oncol.

[122] Dierks C, Seufert J, Aumann K (2021). Combination of lenvatinib and pembrolizumab is an effective treatment option for anaplastic and poorly differentiated thyroid carcinoma. Thyroid.

[123] Liu J, Xiao Q, Xiao J (2022). Wnt/β-catenin signalling: function, biological mechanisms, and therapeutic opportunities. Signal Transduct Target Ther.

[124] Kanematsu R, Hirokawa M, Tanaka A (2021). Evaluation of E-cadherin and β-catenin immunoreactivity for determining undifferentiated cells in anaplastic thyroid carcinoma. Pathobiology.

[125] Wiseman SM, Masoudi H, Niblock P (2006). Derangement of the E-cadherin/catenin complex is involved in transformation of differentiated to anaplastic thyroid carcinoma. Am J Surg.

[126] Bishop JA, Sharma R, Westra WH (2011). PAX8 immunostaining of anaplastic thyroid carcinoma: a reliable means of discerning thyroid origin for undifferentiated tumors of the head and neck. Hum Pathol.

[127] Suzuki A, Hirokawa M, Takada N (2015). Diagnostic significance of PAX8 in thyroid squamous cell carcinoma. Endocr J.

[128] Gulubova M, Ivanova K, Ananiev J (2014). VEGF expression, microvessel density and dendritic cell decrease in thyroid cancer. Biotechnol Biotechnol Equip.

[129] Pavlidis ET, Galanis IN, Pavlidis TE (2023). Update on current diagnosis and management of anaplastic thyroid carcinoma. World J Clin Oncol.

[130] Harris PJ, Bible KC (2011). Emerging therapeutics for advanced thyroid malignancies: rationale and targeted approaches. Expert Opin Investig Drugs.

[131] Kang YE, Kim JT, Lim MA (2019). Association between circulating fibroblast growth factor 21 and aggressiveness in thyroid cancer. Cancers (Basel).

[132] Cabanillas ME, Habra MA (2016). Lenvatinib: role in thyroid cancer and other solid tumors. Cancer Treat Rev.

[133] Giuffrida D, Gharib H (2000). Anaplastic thyroid carcinoma: current diagnosis and treatment. Ann Oncol.

[134] Rusinek D, Chmielik E, Krajewska J (2017). Current advances in thyroid cancer management. Are we ready for the epidemic rise of diagnoses?. Int J Mol Sci.

[135] Mitchell AL, Gandhi A, Scott-Coombes D, Perros P (2016). Management of thyroid cancer: United Kingdom National Multidisciplinary Guidelines. J Laryngol Otol.

[136] Liu R, Xing M (2016). TERT promoter mutations in thyroid cancer. Endocr Relat Cancer.

[137] Landa I, Ibrahimpasic T, Boucai L (2016). Genomic and transcriptomic hallmarks of poorly differentiated and anaplastic thyroid cancers. J Clin Invest.

[138] Bible KC, Kebebew E, Brierley J (2021). 2021 American Thyroid Association guidelines for management of patients with anaplastic thyroid cancer. Thyroid.

[139] Poulikakos PI, Sullivan RJ, Yaeger R (2022). Molecular pathways and mechanisms of BRAF in cancer therapy. Clin Cancer Res.

[140] Prete A, Borges de Souza P, Censi S, Muzza M, Nucci N, Sponziello M (2020). Update on fundamental mechanisms of thyroid cancer. Front Endocrinol (Lausanne).

[141] Manzella L, Stella S, Pennisi MS (2017). New insights in thyroid cancer and p53 family proteins. Int J Mol Sci.

[142] Ibrahimpasic T, Ghossein R, Shah JP, Ganly I (2019). Poorly differentiated carcinoma of the thyroid gland: current status and future prospects. Thyroid.

[143] Wong K, Di Cristofano F, Ranieri M, De Martino D, Di Cristofano A (2019). PI3K/mTOR inhibition potentiates and extends palbociclib activity in anaplastic thyroid cancer. Endocr Relat Cancer.

[144] Pozdeyev N, Rose MM, Bowles DW, Schweppe RE (2020). Molecular therapeutics for anaplastic thyroid cancer. Semin Cancer Biol.

[145] Xu B, Qin T, Yu J, Giordano TJ, Sartor MA, Koenig RJ (2020). Novel role of ASH1L histone methyltransferase in anaplastic thyroid carcinoma. J Biol Chem.

[146] Volante M, Lam AK, Papotti M, Tallini G (2021). Molecular pathology of poorly differentiated and anaplastic thyroid cancer: what do pathologists need to know?. Endocr Pathol.

[147] Araque KA, Gubbi S, Klubo-Gwiezdzinska J (2020). Updates on the management of thyroid cancer. Horm Metab Res.

[148] Garcia C, Buffet C, El Khattabi L (2019). MET overexpression and activation favors invasiveness in a model of anaplastic thyroid cancer. Oncotarget.

[149] Papanikolaou V, Kyrodimos E, Mastronikolis N (2023). Anti-EGFR/BRAF-tyrosine kinase inhibitors in thyroid carcinoma. Cancer Diagn Progn.

[150] Liang M-X, Fei Y-J, Yang K, Tang W-J, Cao X-H, Tang J-H (2022). Potential values of circulating tumor cell for detection of recurrence in patients of thyroid cancer: a diagnostic meta-analysis. BMC Cancer.

[151] Misiak D, Bauer M, Lange J (2021). MiRNA deregulation distinguishes anaplastic thyroid carcinoma (ATC) and supports upregulation of oncogene expression. Cancers (Basel).

[152] Yan K, Liu QZ, Huang RR (2024). Spatial transcriptomics reveals prognosis-associated cellular heterogeneity in the papillary thyroid carcinoma microenvironment. Clin Transl Med.

[153] Han PZ, Ye WD, Yu PC (2024). A distinct tumor microenvironment makes anaplastic thyroid cancer more lethal but immunotherapy sensitive than papillary thyroid cancer. JCI Insight.

[154] Wirth LJ, Sherman E, Robinson B (2020). Efficacy of selpercatinib in RET-altered thyroid cancers. N Engl J Med.

[155] Hadoux J, Elisei R, Brose MS (2023). Phase 3 trial of selpercatinib in advanced RET-mutant medullary thyroid cancer. N Engl J Med.

[156] Subbiah V (2021). Pralsetinib for patients with advanced or metastatic RET-altered thyroid cancer (ARROW): a multi-cohort, open-label, registrational, phase 1/2 study. Lancet Diabetes Endocrinol.

[157] Lee SE, Lee MS, Bang H, Kim MY, Choi YL, Oh YL (2023). NTRK Fusion in a Cohort of BRAF p. V600E Wild-Type Papillary Thyroid Carcinomas. Mod Pathol.

[158] Park JC, Ashok A, Liu C, Kang H (2022). Real-world experience of NTRK fusion-positive thyroid cancer. JCO Precis Oncol.

[159] Michel Ocampo M, Lerner J, Tosonian S, Dasanu CA (2021). Advanced papillary thyroid carcinoma responding to nivolumab. J Oncol Pharm Pract.

